# Strengthening integrated depression services within routine primary health care using the RE-AIM framework in South Africa

**DOI:** 10.1371/journal.pgph.0002604

**Published:** 2023-11-13

**Authors:** Inge Petersen, Christopher G. Kemp, Deepa Rao, Bradley H. Wagenaar, Max Bachmann, Kenneth Sherr, Tasneem Kathree, Zamasomi Luvuno, André Van Rensburg, Sithabisile Gugulethu Gigaba, Londiwe Mthethwa, Merridy Grant, One Selohilwe, Nikiwe Hongo, Gillian Faris, Christy-Joy Ras, Lara Fairall, Sanah Bucibo, Arvin Bhana

**Affiliations:** 1 Centre for Rural Health, School of Nursing and Public Health, University of KwaZulu-Natal, Durban, South Africa; 2 Institute for Global Health, University College London, London, United Kingdom; 3 Department of International Health, Johns Hopkins Bloomberg School of Public Health, Baltimore, MD, United States of America; 4 Department of Global Health, University of Washington, Seattle, WA, United States of America; 5 Norwich Medical School, University of East Anglia, Norwich, Norfolk, United Kingdom; 6 Curtin University, Perth, Australia; 7 Mental Health Directorate, KwaZulu-Natal Department of Health, Pietermaritzburg, South Africa; 8 Knowledge Translation Unit, University of Cape Town, Cape Town, South Africa; 9 SA Medical Research Council, Health Systems Research Unit, Durban, South Africa; University of Liverpool, UNITED KINGDOM

## Abstract

Integration of mental health into routine primary health care (PHC) services in low-and middle-income countries is globally accepted to improve health outcomes of other conditions and narrow the mental health treatment gap. Yet implementation remains a challenge. The aim of this study was to identify implementation strategies that improve implementation outcomes of an evidence-based depression care collaborative implementation model integrated with routine PHC clinic services in South Africa. An iterative, quasi-experimental, observational implementation research design, incorporating the Reach, Effectiveness, Adoption, Implementation and Maintenance (RE-AIM) framework, was applied to evaluate implementation outcomes of a strengthened package of implementation strategies (stage two) compared with an initial evaluation of the model (stage one). The first stage package was implemented and evaluated in 10 PHC clinics and the second stage strengthened package in 19 PHC clinics (inclusive of the initial 10 clinics) in one resource-scarce district in the province of KwaZulu-Natal, South Africa. Diagnosed service users were more likely to be referred for counselling treatment in the second stage compared with stage one (OR 23.15, SE = 18.03, z = 4.04, 95%CI [5.03–106.49], *p* < .001). Training in and use of a validated, mandated mental health screening tool, including on-site educational outreach and technical support visits, was an important promoter of nurse-level diagnosis rates (OR 3.75, 95% CI [1.19, 11.80], *p* = 0.02). Nurses who perceived the integrated care model as acceptable were also more likely to successfully diagnose patients (OR 2.57, 95% CI [1.03–6.40], *p* = 0.043). Consistent availability of a clinic counsellor was associated with a greater probability of referral (OR 5.9, 95%CI [1.29–27.75], *p* = 0.022). Treatment uptake among referred service users remained a concern across both stages, with inconsistent co-located counselling services associated with poor uptake. The importance of implementation research for strengthening implementation strategies along the cascade of care for integrating depression care within routine PHC services is highlighted.

## Introduction

Low- and middle-income countries (LMICs) face a crisis as the high existing toll of communicable diseases meets the rapidly growing burden of non-communicable diseases (NCDs) [1]. This crisis is coupled with a large untreated burden of depression—an estimated 92% of people with depression do not receive treatment [2], with depressive disorders being the most prevalent health condition in sub-Saharan Africa [3]. Depression co-existing with chronic medical conditions is associated with poorer health outcomes and increased mortality [4, 5]. The World Health Organization (WHO) has advocated for integration of treatment for mental health conditions into primary health care (PHC) for decades. In addition to providing a viable approach for narrowing the treatment gap, this approach provides a platform for the treatment of co-existing mental and medical conditions simultaneously, improving outcomes for both [6]. The Mental Health Gap Action Programme (mhGAP), initiated in 2008, offers training, evidence-based guidelines, and tools for assessment and integrated management of mental health conditions by non-specialist PHC practitioners using a task-sharing approach [7]. The collaborative care model has demonstrated strong effectiveness in treating people with mental and physical multi-morbidities, though most of this evidence comes from controlled trials in high- and middle-income countries [8–10]. There is little evidence on best practices for integrated collaborative depression care for people with chronic medical conditions, within routine PHC services, from lower-resource contexts globally [11–13]. This study used implementation science to help narrow this evidence gap in one LMIC country–South Africa.

South Africa provided an opportune country setting to test and understand best practices to implement integrated depression care into routine PHC services for people with chronic conditions for the following reasons. It has a PHC system uniquely burdened by high rates of HIV and TB coupled with rapidly growing rates of NCDs [14], alongside a high burden of undetected co-existing mental health conditions [15, 16]. Against this epidemiological backdrop, health systems reforms aimed at strengthening integrated care for people with chronic conditions at the PHC level provided an enabling policy environment [17, 18].

The aim of this study was to use implementation research methods to strengthen and evaluate a package of implementation strategies to optimise real-world delivery of an evidence-based integrated collaborative depression care model for PHC services in South Africa tested in a cluster randomized trial elsewhere [19]. This original model strengthened nurse clinician assessment, diagnosis and management of comorbid depression in chronic care PHC patients, inclusive of strengthened referral pathways for medical and psychological treatment. The study formed part of the Southern African Research Consortium for Mental health INTegration (S-MhINT).

## Methods

### Study site

The study site was the Amajuba District of the KwaZulu-Natal (KZN) province on the eastern seaboard of South Africa. The district is home to approximately 500 000 people and comprises three sub-districts covering urban, peri-urban and rural areas. It thus provided the opportunity to examine implementation of the collaborative care package across diverse settings. The evidence-based model was initially implemented in 13 fixed-PHC clinics in one urban sub-district, with fixed-clinics comprising infrastructure located in one geographic area. Informed by findings from an evaluation of this initial implementation, the implementation strategy package was then strengthened and expanded to all fixed-PHC clinics across the three sub-districts of Amajuba (n = 24), inclusive of the initial clinics where it was implemented. All PHC clinics were serviced by Professional Nurses who have a three/four-year diploma/degree in nursing, Enrolled Nurses who have a two-year diploma, sessional PHC doctors who visit for one session per week, and HIV counsellors funded by the Department of Health (DoH) using a conditional grant from the United States President’s Emergency Plan for AIDS Relief (PEPFAR). Across the study period (2018–2022), mental health specialist referral services provided by the DoH comprised between one to three clinical psychologists who offered psychological services at two district hospitals.

### Study design

**Fig 1:**
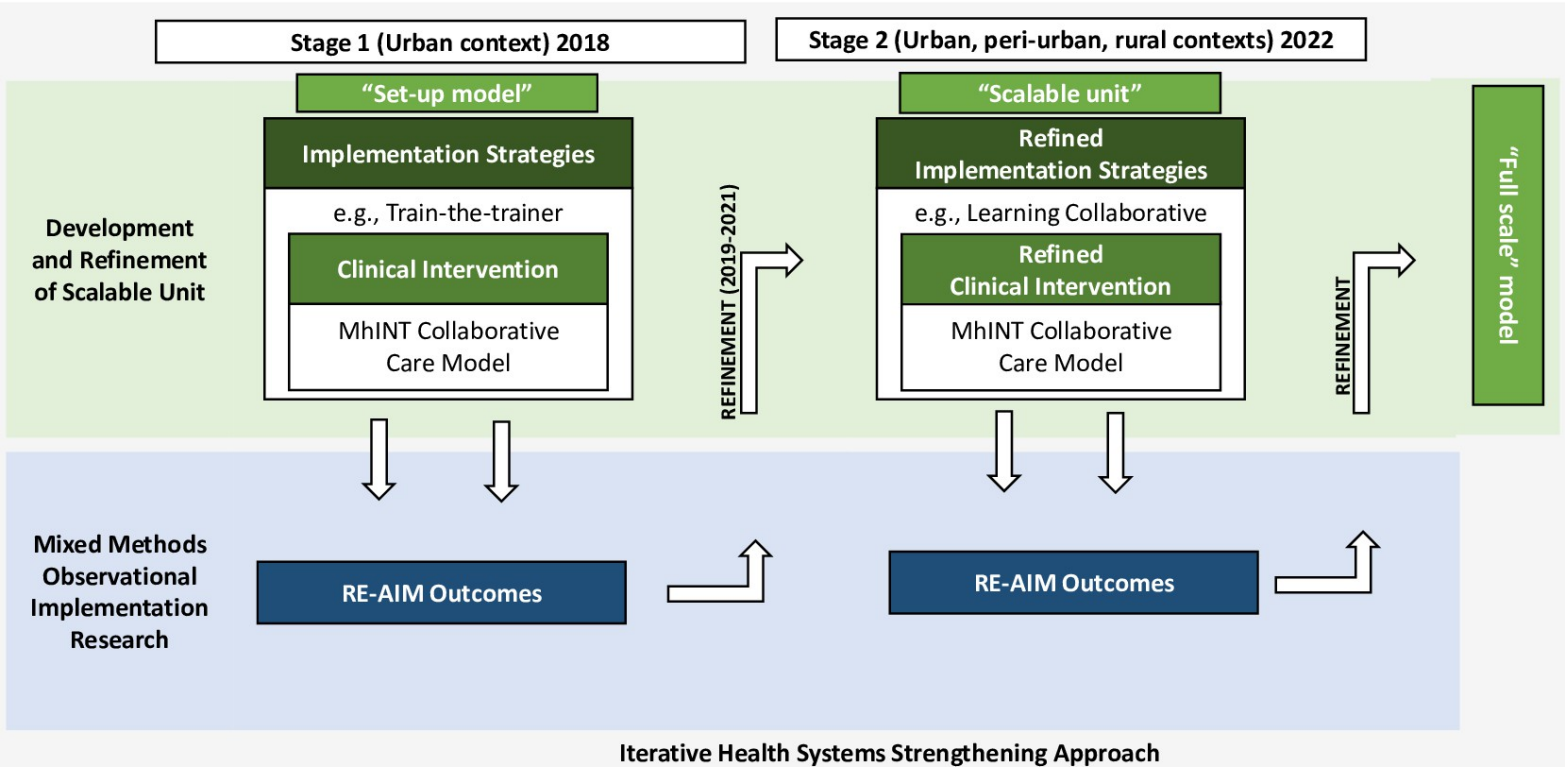
Two stage iterative design.

The study adopted a two-stage, iterative, quasi-experimental, observational mixed methods implementation research design [20], reported in detail in the research protocol [21] (See Fig 1). We used the same study procedures in both stages to minimise bias. In both stages, implementation outcomes were evaluated at the service user, provider, and organizational levels using the RE-AIM (Reach, Effectiveness, Adoption, Implementation, Maintenance) framework [22, 23]. RE-AIM has been widely used to assess the population health impact of interventions in real-world settings.

### RE-AIM data collection and analysis

#### Data measures and collection procedures

To assess RE-AIM outcomes across both stages, we used project data sources, including facility profiles and implementation records, data collected through two patient cohorts, one in stage one and another in stage two; as well as two rounds of a cross-sectional provider survey, also one in each stage. Although all study participants were de-identified using study identification numbers, the Principal Investigator and data manager had access to separately held implementation records containing information with personal identifiers of individual participants during and after data collection to facilitate follow-up of cohort participants, link participants in terms of counselling uptake, and maintain training records to link to provider characteristics and outcomes. Identifying details were deleted once linked. The cohort studies and nurse cross-sectional surveys were conducted in 2018 in 10 of the 13 facilities in the urban sub-district in the first stage; and in the second-stage were conducted in 2022 in 19 of the 24 facilities across the urban, semi-rural and rural districts (inclusive of the original 10 clinics in the urban sub-district). Missing data on key variables from each stage of data collection was dropped for the purpose of analysis. Less than 2% of data in each stage was missing. Non-participation occurred if the nurse was off-duty or on night shift at the time of the data collection. Strengthening of the intervention occurred from 2019–2021, with delays incurred due to the COVID-19 pandemic.

**Reach** was assessed along the cascade of care at the facility level at both stages. We used cohort baseline data from 412 participants from stage one, and 633 participants from stage two, who screened positive for depressive symptoms by our fieldworkers prior to assessment by a nurse. We assessed the proportion and characteristics of participants in these cohorts who were subsequently diagnosed, referred to care, and who took up the co-located counselling services provide by trained and supervised existing HIV clinic-based counsellors. The study samples in both stages were powered based on the cohort design [24]. Nurses were asked to complete a checklist indicating whether they had made a depression diagnosis and a referral to a counsellor in both stages. Participants in the first stage were restricted to adult service users with an existing physical chronic illness. At the request of the DoH, participants in the second stage included all adult PHC service users attending chronic and acute services. We linked research participants referred for counselling to independent project data on counselling attendance records in both stages. This was done manually using clinic and participant name, both of which were collected by the two independent data bases. Generalized linear mixed-effects models were used to estimate associations between patient and facility characteristics and service delivery outcomes along the cascade of care. To compare reach along the cascade of care between the two stages, logistic regression was used to estimate the difference in overall rates of diagnosis, referral and uptake, adjusting for facility clustering and the same covariates used in the stage-specific analyses.

**Effectiveness** was measured in the first stage in terms of associations between PHC nurse diagnosis and referral for depression management and subsequent patient-level depression outcomes using the patient cohort. Cohort participants were identified by screening primary care attendees at the participating PHC facilities for depression symptoms using the Patient Health Questionnaire-9 (PHQ-9) by research assistants. Participants scoring ≥9,—the locally validated cut-off for the chronic patient population [25]–were offered enrolment and followed up at 3- and 9-months post-enrolment in the first stage evaluation. In the second stage, because of delays associated with the COVID-19 pandemic, participants were only followed up at 3-months post-enrolment. In the first stage, a 50% reduction in PHQ-9 raw scores from baseline to 3- and 9-months follow-up were compared across three groups–cohort patients screened positive for depression by our research fieldworkers who were not diagnosed; patients diagnosed with depression but who were not referred for treatment; and patients diagnosed and referred for treatment–while adjusting for baseline PHQ-9 scores and other characteristics using multivariable models [24]. These three groups were identified using the nurse checklist used for the Reach data, where they indicated whether they had made a depression diagnosis and a referral to a counsellor.

**Adoption** focused on assessing and understanding variation in the Reach care cascade at an individual and facility level. In relation to variation in referral and counselling uptake across facilities in the first stage, we used a generalized linear mixed effects model to estimate the association between the availability of a facility counsellor during the cohort implementation period and the proportion of referred service users successfully receiving at least one counselling session at each facility. Facility-specific counts of service users referred for counselling over the implementation period were used as frequency weights. The model included a random facility-specific intercept and used the binomial family and logit link. In the second stage, we applied logistic regression analysis to the baseline cohort data linked with project counselling attendance data and counsellor availability to assess variations in facility rate of referral and counselling uptake of service users screening positive for depression. To understand characteristics of nurse providers with high and low referral rates, we administered a cross-sectional survey during the first and second stage cohort studies to the full complement of available Nurse Clinicians servicing the facilities in the first (N = 68) and second stages (N = 136). These data were linked with nurse self-reported referrals on the nurse checklist that was used to identify high and low referring nurses, determined by whether nurses reported 100% of screen positive patients referred vs. less than 100%. At the time of the study, nurse clinicians in South Africa were not mandated to provide anti-depressant treatment and did not have the time or capacity to provide counselling. Unlike in other contexts, referral presented the only avenue for accessing treatment for patients diagnosed with depression. The quality metric of 100% for referral was thus used given this context.

The survey included the following scales previously administered in South Africa: Organizational Readiness for Implementing Change (ORIC), the MICA Mental Illness: Clinicians’ Attitudes Scale (MICA), Role Overload Scale and Intervention Acceptability (AIM) (See Kemp, Mntambo et al. 2021 for further details) [26] in both stages. The General Health Questionnaire (GHQ) was added to assess provider physical and mental health status in the second stage. Project records on screening outreach training as well as online nurse training records were also linked to the nurse survey data in the second stage. We applied logistic regression to determine predictors of nurse variation in diagnosis and referral.

For **Implementation**, we assessed whether consistency of facility-counsellor presence was associated with counselling referrals and counselling uptake. We used project record data for January to December 2018 and January to September 2022. Referral rates were extracted from referral forms and patient counselling uptake was assessed using patient tracking forms. Consistency of lay-counsellor presence was assessed using facility profiles. Nurse checklist data on referrals from the cohort study were linked with project record data on counselling uptake via patient names as well as counsellor presenteeism from facility profiles during the cohort study period. In the first stage, we used a generalized linear mixed effects regression model to estimate the association between the availability of a facility counsellor throughout the implementation period and the proportion of referred service users successfully receiving at least one counselling session at each facility. In the second stage, we added consistency of lay counsellor presence to the baseline cohort data and used generalized linear mixed-effects models to estimate associations between patient counselling uptake and counsellor presenteeism.

We assessed counselling fidelity using a bespoke counselling fidelity rating checklist adapted from ENACT and previously used as part of the evaluation of the original package [27].

For **Maintenance**, at the individual level, we followed up service users in the first stage cohort study at 9 months to establish stability of effects on depression symptom reduction over time. At the organizational level, we reviewed minutes of provincial meetings with policy makers from the Mental Health Directorate of the KZN DoH as well as meetings held with national policy makers to assess uptake of innovations from the SMhINT implementation package into policy.

#### Ethical considerations

Fieldworkers verbally explained the rationale for the study to potential participants. Once verbal consent was given, the study details were explained, and questions from the potential participants were addressed. The voluntary nature of participation was reiterated, and willing participants provided informed signed consent. Participants were given a copy of the information document for their records. The study was performed in accordance with the ethical standards contained in the 1964 Declaration of Helsinki. Ethical approval was obtained from the Biomedical Research Ethics Committee at the University of KwaZulu-Natal (BF190/17) and the Directorate of Health Research and Knowledge Management in KwaZulu-Natal (HRKM253/17 KZ_2017RP15_388) in the KwaZulu-Natal Department of Health.

### Description of the implementation package and how the results of the first stage evaluation informed the strengthening of the implementation strategies in the second stage

The initial and strengthened packages of implementation strategies were layered onto existing activities for other conditions within PHC facilities and included several strategies from the Expert Recommendations for Implementing Change (ERIC) compilation [28] (italicised below). The initial package aimed to i) Orientate and optimize buy-in and support from district and PHC clinic managers through orientation workshops (*educational meetings and preparing champions*); ii) Improve *demand* through training (*educational meetings*) of existing staff to provide psychoeducational material on depressive symptoms in the waiting room area so as to strengthen mental health literacy; iii) Improve nurse clinician assessment and diagnosis of depressive symptoms through the provision of additional mental health training (using a *train the trainer* approach) in the use of an existing national evidence-based clinical decision support tool aligned with mhGAP called Adult Primary Care (APC)—also known internationally as the Practical Approach to Care Kit (PACK) [29]; iv) Strengthen referral pathways through training (*educational meetings*) and *supervision* of existing facility-based HIV counsellors in a manualized lay-counselling intervention (*educational materials*) that drew on cognitive behavioral techniques and problem solving, both shown to be effectively delivered using a task sharing approach [30]. This implementation strategy package complemented existing mental health services that included i) Screening provided in the vital signs room, although there was no standardized, validated screening tool in use, with PHC clinics adopting a variety of tools made up of screening items from various instruments; ii) Existing referral pathways for people with more severe depressive symptoms to the sessional PHC doctors to whom initiation of antidepressant medication was restricted, or to mental health specialists at the district level. Project-supported *technical support* using *cyclical small tests of change* was used to embed the different components within the system, using routine mental health indicators of number screened and number treated. The first stage package is outlined in Table 1, with more details available in previous publications [21, 31].

**Table 1:** Summary of provider levels, intervention components, implementation strategies, and implementation challenges at each evaluation stage.

Provider	Intervention	First Stage Implementation Strategies	Challenges Identified in First Stage	Second Stage Strengthened Implementation Strategies
Provincial, District and Facility managers Researchers	Promote acceptance and buy-in of integrated depression care	*Develop a formal implementation blueprint* *Conduct educational meetings* (Orientation workshops) *Identify and prepare champions* (Orientation workshops) *Cyclical small tests of change*	Health care providers less willing to implement in the absence of the package being mandated by the Department of Health. Paucity of mental health indicators to monitor implementation along the cascade of care.	*Update an implementation blueprint* *Conduct educational meetings* (Orientation workshops) *Identify and prepare champions* (Orientation workshops) *Establish a learning collaborative* (Provincial and district) • *Develop academic partnerships* • *Conduct local consensus discussions* • *Purposely re-examine the implementation* • *Mandate change* • *Tailor strategies* *Revise professional roles* (training of HIV counsellors to become social auxiliary workers)
Health Promoter/CHW	Strengthen patient mental health literacy and counselling demand	*Increase demand* *Develop educational materials* *Distribute educational materials* (Psychoeducational morning talk guidelines on depression developed and distributed)	Poor demand/perceived need for mental health services by service users	*Increase demand* *Develop educational materials including digitalized materials* *Distribute educational materials* *Conduct educational meetings* (Expansion of psychoeducational morning talk guidelines and posters to include grief and bereavement and anxiety. Inclusion of links to self-help skills building videos).
Enrolled Nurse/Professional Nurse	Screening	None (utilized existing screening tools)	Lack of a validated standardized screening tool resulting in inadequate and inconsistent screening	*Develop and implement tools for quality monitoring* [Validated Brief Mental Health (BMH) screening tool and Standard Operating Procedure] *Mandate change* (BMH adopted into policy as the standardized mental health PHC clinic screening tool for KZN) *Develop educational materials* (Training manual) *Educational outreach visits* (Clinic staff trained on-site in use of the tool) *Provide local technical assistance* (District Mental health coordinator supported by project implementation coordinator provided on-site training and technical support)
Professional Nurse	Strengthen capacity to use DoH APC guidelines to diagnose and manage patients with comorbid depression.	*Develop educational materials* *Distribute educational materials* *Conduct educational meetings* *Train the trainer strategy*	Train the trainer approach to capacitate CNPs in the APC mental health guidelines and CNP personal emotional issues limited professional nurse perceived competency and willingness to diagnose and referral of depression in service users	*Develop digital self-directed educational materials* (for APC mental health content and wellness) *Distribute educational materials* (for APC mental health content and wellness) *Provide local technical assistance* (Train a PN to provide technical support for APC online training in each facility) *Audit and feedback* (Information of PN completion of APC course feedback to facilities to improve training coverage)
HIV counsellors/social auxiliary workers	Capacitate existing HIV counsellors/social auxiliary workers to provide manualized depression counselling	*Develop educational materials* *Distribute educational materials* *Conduct educational meetings* *Provide clinical supervision*	Depression counselling not part of the role and functions of existing HIV counsellors leading to variable availability of the counselling service	*Develop educational materials* *Distribute educational materials* *Conduct educational meetings* *Provide clinical supervision* *Create/change credentialing and/or licensure standards* (Training of HIV counsellors to become social auxiliary workers certified to provide limited mental health counselling) *Revise professional roles* (training of HIV counsellors to become social auxiliary workers)

Evaluation of implementation outcomes and determinants of the initial stage one package using the RE-AIM framework are reported in previous publications [24, 26, 32], summarized in Box 1, and outlined in Table 2.

**Box 1. Implementation outcomes and implementation challenges emerging from the first stage evaluation**

**Reach:** Of the sub-sample of cohort participants who screened positive for depressive symptoms prior to assessment by a nurse, 50.5% were diagnosed with depression using APC. Of these, 36.5% were referred, translating into 18.4% of the original cohort sub-sample that screened positive for depressive symptoms at baseline. Of those referred, 23.7% received at least one counselling session, meaning that of the original sub-sample of service users screening positive for depressive symptoms, only 4.4% received at least one counselling session. See Kemp et al. 2021 for more details [26]. Participants more likely to be diagnosed with depression by nurses reported greater symptom severity, suicidal thoughts, perceived stress, disability and high risk/dependent alcohol use details. Participants more likely to be referred had fewer chronic conditions, with the exclusion of HIV; and participants in receipt of at least once counselling session were more likely to have less social support [26].

**Effectiveness**: As reported in Kathree et al. [24], participants diagnosed and referred had lower PHQ-9 scores at three months, and were more likely to have their PHQ-9 scores decreased by more than 50% from baseline to three months follow-up compared to participants diagnosed and not referred [23]. Outcomes did not differ significantly between the groups at nine months follow-up, similar to what was found in a previous evaluation of the same counselling intervention under controlled conditions [33]. As suggested by Kathree et al. [24] this is likely to be a result of spontaneous remission, especially within the mild to moderate cases which were dominant in the sample, and where people with mild to moderate symptoms more likely to spontaneously remit [34].

**Adoption**: At the individual level, project record data over a period of three months during 2018 (June-August 2018) revealed that Professional Nurses referred on average 1.2 service users per month, with median referral rates per month ranging from 0 to 2.75 [26]. Poor referral was attributed to lack of perceived competency—whereby the pre-COVID cascade model of APC training using a train the trainer strategy (whereby master trainers are equipped to train facility trainers who in turn train clinic staff) impacted negatively on implementation fidelity; unattended personal emotional issues of staff; low demand for counselling on the part of service users; and the need for mandating of a standardized and valid routine mental health screening tool [26].

**Implementation**: Half of the clinics in the first stage had a counsellor available over the entire implementation period. Among clinics without a counsellor available over the entire implementation period, 16% (95%CI [1%-30%]) of referred service users received at least one session of counselling. Among facilities with a counsellor available over the entire implementation period, 57% (95%CI [34%- 81%]) received at least one session of counselling. Uptake of the co-located counselling service was compromised by lack of availability of lay counsellors, with availability of a counsellor over the entire implementation period associated with a 42% relative increase in the probability of referred service users receiving at least one counselling session 95%CI [14%- 69%].

Fidelity ratings by the counsellor supervisor using a bespoke fidelity counselling rating scale in the first stage were high across the facilities, ranging from 78 to 98%, with an average of 86%.

**Table 2:** RE-AIM variables, data sources, and results across study stages.

Variable	Source	Results (Stage 1)	Results (Stage 2)
**Reach at the facility level (Individual-level analyses of the proportion and characteristics of the target population that received the intervention along the cascade of care) 1**^**st**^ **stage (10 facilities) and 2**^**nd**^ **stage (20 facilities)**			
% of chronic care patients screening positive for depression at facility level	Cohort data (1^st^ and 2^nd^ stage)	N = 559/1404 (39.7%)*	N = 633/1274 (49.7%)
% of sub-sample of diagnosed chronic care patients screening positive for depression	Cohort data (1^st^ and 2^nd^ stage)	N = 208/412 (50.5%)*	N = 306/633 (48.3%)
Characteristics of chronic care patients diagnosed with depression	Cohort data (1^st^ and 2^nd^ stage)	More likely to have*: •Greater symptom severity •Suicidal thoughts •High risk/dependent alcohol use •Greater perceived stress •Greater disability	More likely to have: •Greater symptom severity •Suicidal thoughts •High risk/dependent alcohol use •Greater perceived stress
% of chronic care diagnosed patients referred	Cohort data (1^st^ and 2^nd^ stage)	N = 76/208 (36.5%)*	N = 279/306 (91.2%)
% of sub-sample of chronic care patients screened positive, diagnosed and referred for lay counselling	Cohort data	N = 76/412 (18.4%)*	N = 279/633 (44.1%)
Characteristics of chronic care patients screening positive for depression who were diagnosed and referred for lay counselling	Cohort data	More likely to have* •Fewer chronic conditions (excluding HIV)*	No significant findings
% of chronic care patients referred for counselling who receive at least one counselling session	Cohort data, project records	Once-off N = 18/76 (23.7%)*	Once-off N = 50/279 (17.9%)
% of sub-sample of chronic care patients screening positive who were diagnosed, referred and received at least once counselling session	Cohort data, project records	N = 18/412 (4.4%)*	N = 50/633 (7.9%)
Characteristics of referred patients taking up one or more counselling sessions	Cohort data; CFIR interviews with patients receiving one or more counselling sessions and those receiving no sessions.	Patients taking up counselling more likely to have*: •Less social support	Patients taking up counselling more likely to: •Have completed high school
**Effectiveness (Real-world effectiveness on patient-level outcomes)**			
Depressive symptoms	Patient cohorts	Diagnosed and referred patients more likely to have a 50% reduction in PHQ9 compared to diagnosed and not referred (adjusted odds ratio 2.07, 95% CI 1.12, 3.35, P = 0.03)***	N/A
**Adoption (Organizational-level outcome referring to the proportion and characteristics of settings/service providers that adopt the intervention)**			
Average and median number of patient participants referred for depression care.	Cohort project record data	CNPs referred on average 1.2 patients per month (median: 0.5, range: 0 to 11.5) over a 3-month period.	CNPs referred on average 2 patients per month (median: 1.5, range: 0.5 to 8.5) over a four-month period.
Characteristics of providers who diagnosed and referred one or more patients with depression	Cross sectional survey using ORIC; MICA; AIM; Perceived competency; Training exposure (2^nd^ stage only); GHQ (2^nd^ stage only) Qualitative interviews (1^st^ Stage only)	High referring providers more likely to have: •Higher perceived competency**	High diagnosing and referring providers more likely to have: •Received training in BMH (OR 3.75, 95% CI [1.19, 11.80] *p* = 0.02)]) (Diagnosis) •Higher perceived acceptability of the intervention (OR 2.57, 95% CI [1.03–6.400], *p* = 0.043) (Diagnosis)
**Implementation (Extent to which the intervention was implemented with consistency and fidelity along the cascade of care, adaptations made during the study, and cost)**			
Consistent presence of a facility counsellor	Facility Profiles	50% of cohort clinics	68.4% of cohort clinics
Characteristics of facilities with higher/lower rate of referral and uptake of one or more counselling sessions	Cohort data linked with facility profiles during the cohort study periods	Availability of a counsellor over the entire cohort study period was associated with a 42% relative increase in the probability of referred patients receiving at least one counselling session 95% CI [14–69].	Availability of a counsellor over the entire cohort study period was associated with a greater probability of being diagnosed and referred (OR 5.9, 95%CI [1.29–27.75], *p* = 0.022).
Fidelity of counselling intervention	Fidelity checklists;	Range: 78 to 98%, with an average of 86%	Data not available
**Maintenance (Organizational-level institutionalization of the programme over time, as well as the individual-level sustainability of health outcomes)**			
Stability of effects of the intervention on patient level outcomes of effectiveness over time	Patient Cohort	Outcomes did not differ significantly between groups at nine months***	Not measured
Institutionalization of the intervention within the health care system	Minutes of national and provincial meetings		CMED tool accepted as the mental health tool to be used by CHWs both by the national DOH and the KZN DOH. Psychoeducational material scaled up to all districts in the KZN province by the KZN DOH. BMH tool accepted as the standardized mental health screening tool to be used in PHC facilities by the KZN DOH. APC integrated guidelines and online 30 session APC training with additional mental health modules available nationally.

*Details of analysis and results: Kemp et al., 2020 [32]

**Details of analysis and results: Kemp et al., 2021 [26]

*** Kathree et al., 2023 [24]

### Strengthening of the implementation strategy package in response to the findings of the first stage evaluation

Findings from the first stage evaluation were shared as they emerged and *implementation was purposely re-examined* collectively through *local consensus discussions* to address the challenges identified to *tailor implementation strategies*, *mandate changes* at a policy level including *tools*, *record systems and roles of healthcare providers*. This process also responded to additional mental health issues emerging during the COVID-19 pandemic.

The second stage package is also outlined in Table 1, with more details provided in online supplements using the Template for Intervention Description and Replication (TIDieR) checklist [35]. The extended TIDieR prompts researchers to describe the intervention as well as specify implementation strategies [36]. Similar to Proctor et al. guidance [37], the checklist includes specification of who provided each strategy (who enacted), how (actions/activities to support implementation), to whom (action target/entity impacted), and when and how much (temporality and dose) [35].

The challenges and changes are summarized below and outlined in Table 1:

- **Poor demand for mental health services by service users**
  The *educational material* and *education workshops* for providers of waiting room talks were expanded beyond just depressive symptoms, to include anxiety and grief and bereavement, particularly related to COVID-19. Greater emphasis was placed on capacitating service users with skills to cope with life stressors, beyond healthy lifestyle information included in the first stage. To this end, links to *digitalized educational materials* using cognitive behavioural, problem solving and mindfulness skills-building animated videos were provided as well as toll-free counselling hotline telephone numbers. Educational materials were *distributed* across all facilities in the district (See more details in TIDieR description in the online Supporting Information, S1 Appendix)
- **Lack of a validated standardized screening tool resulting in inadequate and inconsistent screening**
  A standardized screening tool was developed and validated to ensure quality of screening (*develop and implement tools for quality monitoring*) [Brief Mental Health (BMH) screening tool] [38]. The use of the tool was *mandated* through adoption into policy by the KZN DoH. A training manual (*training material)* and standard operating procedure (*develop and implement tools for quality monitoring*) for use of the tool was co-developed with the KZN DoH. It was initially used alongside screening for other conditions within PHC facilities in ‘vital signs’ rooms by Enrolled Nurses where service users are triaged clinically before consultations, but later also extended for use by Professional Nurses. Training of Operational Managers, Professional Nurses and Enrolled Nurses in each PHC clinic was delivered through *educational outreach visits* that also included *small tests of change* provided by the District Mental Health coordinator and S-MhINT project implementation coordinator (*technical assistance*) (See more details in TIDieR description in the online Supporting Information, S2 Appendix).
- **Train the trainer approach to capacitate Professional Nurses in the APC mental health guidelines and nurse healthcare worker emotional burden limited perceived competency and willingness to diagnose and refer depression in service users**
  With the start of the COVID-19 pandemic, APC training was moved to a digital platform instead of an in-person, train-the-trainer approach. *Digitalized educational material* was developed and *distributed* to all Professional Nurses (with no mobile data-related costs to users). The role of the clinic facility trainer was re-configured to provide *technical assistance* for uptake of self-directed education. This was because more than 80% of the nurses had no experience of online training, and required assistance with registration on the platform [39]. Shifting to digital training ensured fidelity of delivered training material and facilitated tracking of training coverage in each facility. These data were fed back to each facility to improve training coverage (*audit and feedback*). In addition, digitalized wellness *educational material* was developed for PHC nurses to support coping with the stressors imposed by COVID-19 and the emotional labour accompanying assessment, diagnosis and management of people with mental health conditions. (See more details in TIDieR descriptions in the online Supporting Information, S3 and S4 Appendices
- **Depression counselling not part of the role and functions of existing HIV counsellors leading to variable availability of the counselling service**
  HIV counsellors who were being trained by the KZN DoH to become social auxiliary workers were targeted for training to improve role consonance (*revise credentialing and roles*) with their job description (See more details in TIDieR description in the online Supporting Information, S5 Appendix

## Results

Stage two RE-AIM outcomes are summarised and compared against stage one outcomes in Table 2.

### Reach

In the second stage, a sample of 1274 participants consented to be screened for depressive symptoms and participate in the cohort study, with a 3% refusal rate. Of the sub-sample of 633 stage two cohort participants who screened positive for depressive symptoms prior to assessment by a nurse, 48.3% were diagnosed with depression by nurses (N = 306/633). Of these, 91.2% were referred (N = 279/306) translating into 44.1% (N = 279/633) of the cohort sub-sample that screened positive for depressive symptoms at baseline being referred for counselling. However, of those referred, only 17.8% received at least one counselling session (N = 50/279), translating into 7.9% of the original sub-sample of service users screening positive for depressive symptoms (N = 50/633). (Table 2)

Participants who were more likely to be diagnosed with depression by nurses were more likely to report greater symptom severity (OR 1.07 95%[CI 1.00–1.15], *p* = 0.048), suicidal thoughts (OR 1.69 95%[CI 1.04–2.76] *p* = 0.035), perceived stress (OR 1.08 95%[CI 1.03–1.12] *p* = 0.001), and high risk/dependent alcohol use (OR 1.12 95%[CI 1.01–1.23] *p* = 0.035), similar to the first stage. Unlike the first stage, most of the diagnosed service users were also referred (91.2%), so it was not possible to identify patient-level characteristics associated with referral in the second stage. Patient participants receiving at least once counselling session were less likely to have completed high school compared to those that had (OR 0.24 95% [CI 0.07–0.79] *p* = 0.02). (See Tables 3 and 4).

**Table 3:** Stage two patient cohort participant characteristics and associations with diagnosis, referral and uptake of counselling (n = 633).

	DETECTION			REFERRAL (given detection)			> = ONE SESSION UPTAKE (given referral)			
Factor	Not Detected	Detected	*p* ^*1*^	Not Referred	Referred	*p* ^*1*^	No Uptake	Uptake	*p* ^*1*^	TOTAL
N	327	306		27	279		229	50		633
Age, mean (SD)	37.4 (13.3)	39.6 (13.1)	0.07	36.4 (10.0)	39.9 (13.4)	0.22	39.5 (13.6)	41.8 (12.0)	0.04	38.5 (13.3)
Female	257 (78.6%)	248 (81.0%)	0.44	22 (81.5%)	226 (81.0%)	0.94	190 (83.0%)	36 (72.0%)	0.001	505 (79.8%)
Black	325 (99.4%)	302 (98.7%)	0.49	27 (100.0%)	275 (98.6%)	*	226 (98.7%)	49 (98.0%)	0.68	627 (99.1%)
Completed Grade 12	121 (37.0%)	111 (36.3%)	0.88	12 (44.4%)	99 (35.5%)	0.39	88 (38.4%)	11 (22.0%)	014	232(36.7%)
Employed	70 (21.4%)	66 (21.6%)	0.97	8 (29.6%)	58 (20.8%)	0.18	44 (19.2%)	14 (28.0%)	0.15	136 (21.5%)
Low Income (2000 ZAR/ month)	229 (81.5%)	210 (82.0%)	0.90	17 (77.3%)	193 (82.5%)	0.50	166 (85.1%)	27 (69.2%)	0.05	439 (81.8%)
Household food insecurity										
Healthcare use -3 months prior:	197 (60.2%)	204 (66.7%)	0.22	17 (63.0%)	187 (67%)	0.72	159 (69.4%)	28 (56.0%)	0.05	401 (63.3%)
Other PHC										
Hospitalized										
Number of Co-morbidities under treatment:	216 (66.1%)	184 (60.1%)	0.23	13 (48.1%)	171 (61.3%)	0.10	137 (59.8%)	34 (68.0%)	0.50	400 (63.2%)
0	11 (3.4%)	12 (3.9%)	0.74	2 (7.4%)	10 (3.6%)	0.22	9 (3.9%)	1 (2.0%)	0.46	23 (3.6%)
1										
≥ 2			0.01			0.88			0.96	
Prior depression/ anxiety	123 (37.6%)	79 (25.8%)		6 (22.2%)	73 (26.2%)		59 (25.8%)	14 (28.0%)		202 (31.9%)
PSS	170 (52.0%)	177 (57.8%)		17 (63.0%)	160 (57.3%)		132 (57.6%)	28 (56.0%)		347 (54.8%)
Low stress	34 (10.4%)	50 (16.3%)		4 (14.8%)	46 (16.5%)		38 (16.6%)	8 (16.0)		84 (13.3%)
Moderate stress	19 (5.8%)	36 (11.8%)	0.001	5 (18.5%)	31 (11.1%)	0.36	24 (10.5%)	7 (14.0%)	0.55	55 (8.7%)
High stress										
			<0.001			0.15			0.41	
OSLO Social Support	39 (11.9%)	15 (4.9%)		2 (7.4%)	13 (4.7%)		12 (5.3%)	1 (2.0%)		54 (8.5%)
Poor support	253 (77.4%)	203 (66.6%)		22 (81.5%)	181 (65.1%)		146 (64.0%)	35 (70.0%)		456 (72.2%)
Moderate support	35 (10.7%)	87 (28.5%)		3 (11.1%)	84 (30.2%)		70 (30.7%)	14 (28.0%)		122 (19.3%)
Strong support										
ISMI, range 1–44, mean (SD)			0.04			0.007			0.39	
PHQ-9	166 (50.8%)	180 (58.5%)		10 (37.0%)	170 (60.9%)		135 (59.0%)	35 (70.0%)		346 (54.7%)
Moderate depress	131 (40.1%)	92 (30.1%)		14 (51.9%)	78 (28.0%)		66 (28.8%)	12 (24.0%)		223 (35.2%)
Moderately severe	30 (9.2%)	34 (11.1%)		3 (11.1%)	31 (11.1%)		28 (12.2%)	3 (6.0%)		64 (10.1%)
Severe depression	25.74 (6.17)	27.65 (7.16)	0.15	25.00 (8.73)	27.91 (6.95)	0.12	27.55 (7.14)	29.56 (5.78)	0.10	26.67 (6.73
Any suicidal thoughts										
AUDIT-C > 0			<0.001			0.87			0.77	
Counsellor available	277 (84.7%)	208 (68.0%)		20 (74.1%)	188 (67.4%)		157 (68.6%)	31 (62.0%)		485 (76.6%)
	39 (11.9%)	68 (22.2%)		5 (18.5%)	63 (22.6%)		50 (21.8%)	13 (26.0%)		107 (16.9%)
	11 (3.4%)	30 (9.8%)		2 (7.4%)	28 (10.0%)		22 (9.6%)	6 (12.0%)		41 (6.5%)
	72 (22.0%)	131 (42.8%)	<0.001	10 (37.0%)	121 (43.4%)	0.66	94 (41.0%)	27 (54.0%)	0.65	203 (32.1%)
	99 (30.3%)	100 (32.7%)	0.60	11 (40.7%)	89 (31.9%)	0.10	69 (30.1%)	20 (40.0%)	0.06	199 (31.4%)
	257 (78.6%)	242 (79.1%)	0.948	15 (55.6%)	227 (81.4%)	0.023	177 (77.3%)	50 (100.0%)	*	499 (78.8%)

*p*^*1*^ values adjusted for clustering by health facility

*covariate dropped from model due to co-linearity.

PSS: Perceived Stress Scale; OSLO: Oslo Social Support Scale; ISMI: Internalized Stigma of Mental Illness Scale; PHQ-9: Patient Health Questionnaire (9); AUDIT-C: Alcohol Use Disorders Identification Test-C

**Table 4:** Independent associations between participant characteristics and diagnosis, referral and uptake of counselling: Logistic regression mixed* models.

	DIAGNOSED			REFERRED			UPTAKE		
Predictors	Odd Ratios	CI	*p*	Odds Ratios	CI	*p*	Odds Ratios	CI	*p*
Intercept	0.00	0.00–0.05	<0.001	0.81	0.00–186.20	0.940	0.00	0.00 –Inf	0.989
Age	1.03	1.01–1.05	0.006	1.02	0.97–1.08	0.456	1.03	0.98–1.07	0.252
Female	1.90	1.07–3.38	0.029	0.78	0.18–3.40	0.736	1.01	0.34–2.98	0.987
Grade 12	1.44	0.90–2.30	0.129	0.59	0.18–1.91	0.380	0.24	0.07–0.79	0.020
Employed	1.16	0.68–1.98	0.583	0.49	0.12–2.02	0.324	1.86	0.66–5.28	0.242
Low Income	0.91	0.52–1.60	0.744	0.85	0.21–3.48	0.818	0.49	0.15–1.56	0.226
(2000 ZAR/ month)									
Household food	0.91	0.56–1.45	0.680	1.01	0.26–3.90	0.991	0.42	0.15–1.17	0.098
Insecurity									
Other PHC visits	0.74	0.46–1.20	0.220	1.60	0.45–5.69	0.465	1.42	0.53–3.77	0.482
3 months prior									
Hospitalized 3	0.33	0.10–1.08	0.068	0.77	0.06–10.88	0.850	0.00	0.00 –Inf	0.996
months prior									
Comorbidities under	1.88	1.13–3.15	0.015	0.73	0.16–3.37	0.684	0.33	0.10–1.15	0.081
Treatment 1									
Comorbidities under									
Treatment ≥ 2	1.79	0.81–3.96	0.150	0.42	0.05–3.35	0.412	0.31	0.06–1.59	0.159
Prior depression/	1.52	0.73–3.15	0.258	0.25	0.06–1.05	0.059	0.97	0.22–4.18	0.966
Anxiety treatment									
PSS	1.08	1.03–1.12	0.001	1.10	0.97–1.23	0.134	1.01	0.92–1.11	0.807
OSLO	1.00	0.90–1.10	0.975	0.99	0.78–1.27	0.963	0.93	0.76–1.14	0.483
ISMI	1.03	0.98–1.08	0.295	0.98	0.87–1.10	0.712	1.06	0.96–1.17	0.274
PHQ-9	1.07	1.00–1.15	0.048	1.07	0.91–1.26	0.424	1.03	0.90–1.17	0.680
Suicidal Thoughts	1.69	1.04–2.76	0.035	0.45	0.13–1.60	0.219	1.54	0.55–4.30	0.407
AUDIT-C	1.12	1.01–1.23	0.035	0.84	0.68–1.05	0.128	1.06	0.89–1.26	0.483
Counsellor available	1.00	0.35–2.89	0.999	5.99	1.29–27.75	0.022	47529733.48	0.00 –Inf	0.991
Observations	536			255			233		
Facilities	19			19			19		
Marginal R^2^	0.203			0.273			0.931		
Conditional R^2^	0.348			0.394			0.949		

PSS: Perceived Stress Scale; OSLO: Oslo Social Support Scale; ISMI: Internalized Stigma of Mental Illness Scale; PHQ-9: Patient Health Questionnaire (9); AUDIT-C: Alcohol Use Disorders Identification Test-C

* facility as random effect

**Fig 2:**
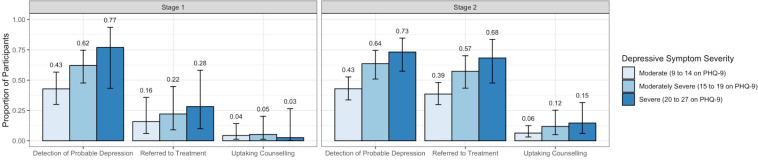
Reach cascade: First and second stage.

Comparative analysis of reach along the cascade of care across both stages was conducted using logistic regression. The logistic regression showed an odds ratio of 21 and 23 (unadjusted and adjusted for covariates, respectively) (OR 23.15, SE = 18.03, z = 4.04, 95%CI [5.03–106.49] *p* < .001). There was no statistically significant difference in the odds of diagnosis and uptake (Fig 2).

### Effectiveness

Given that almost all of the participants who were diagnosed were also referred in the second stage, there were insufficient numbers of patients in the diagnosed and not referred group to conduct an analysis comparing participant PHQ-9 outcomes between diagnosed and not referred and diagnosed and referred groups, as in stage one.

### Adoption

With regard to adoption by Professional Nurses, project record data over a period of four months during 2022 (Jan-April 2022) revealed that Professional Nurses referred on average of two service users per month (median: 1.5), with median referral rates per month ranging from 0.5 to 8.5. Most nurse providers making a diagnosis also made a referral (91.5%), with diagnosing nurse providers more likely to have received training in screening using the BMH (OR 3.75, 95% CI [1.19, 11.80], *p* = 0.02). Diagnosing nurse providers were also more to perceive the intervention as acceptable (OR 2.57, 95% CI [1.03–6.40], *p* = 0.043). (See Tables 5 and 6).

**Table 5:** Stage two cross-sectional provider survey participant characteristics and associations with diagnosis and referral (n = 136).

Factor	Overall	No diagnosis	Diagnosis	p-value
N	136	64	72	
Clinics				0.017
Female	117 (86.0%)	56 (87.5%)	61 (84.7%)	0.64
Age, mean (SD)	47.4 (9.7)	47.7 (19.6)	47.2 (9.0)	0.78
Black	132 (97.1%)	62 (96.9%)	70 (97.2%)	0.90
Married	61 (44.9%)	35 (54.7%)	26 (36.1%)	0.03
Diploma	115 (84.6%)	53 (82.8%)	62 (86.1%)	0.60
Job Title:				0.17
Facility Manager	16 (11.8%)	11 (17.2%)	5 (6.9%)	
Other	6 (4.4%)	4 (4.7%)	3 (4.2%)	
Registered Nurse or Nursing Sister	114 (83.8%)	50 (78.1%)	64 (88.9%)	
Years working, mean (SD)	20.5 (10.1)	20.1 (10.7)	20.9 (9.7)	0.66
Years at clinic, mean (SD)	7.4 (6.2)	6.5 (5.8)	8.2 (6.5)	0.12
Overtime	34 (26.0%)	19 (30.6%)	15 (21.7%)	0.25
Nightshift	17 (12.5%)	13 (20.3%)	4 (5.6%)	0.009
MICA, mean (SD)	41.5 (9.8)	43.2 (10.2)	40.0 (9.3)	0.059
ACCEPTABILITY, mean (SD)	4.2 (0.5)	4.1 (0.4)	4.3 (0.5)	0.013
ORIC Commitment, mean (SD)	3.9 (0.7)	3.8 (0.8)	3.9 (0.6)	0.17
ORIC Efficacy, mean (SD)	3.9 (0.7)	3.8 (0.7)	3.9 (0.6)	0.29
ORIC, mean (SD)	3.9 (0.7)	3.8 (0.7)	3.9 (0.6)	0.22
Role Overload, mean (SD)	3.1 (0.7)	3.1 (0.7)	3.1 (0.7)	0.70
GHQ, mean (SD)	0.9 (0.5)	0.9 (0.5)	0.9 (0.5)	0.77
APC Full Complete	42 (53.8%)	18 (54.5%)	24 (53.3%)	0.92
APC Wellness Complete	3 (2.9%)	1 (2.1%)	2 (3.5%)	0.66
BMH training	108 (79.4%)	44 (68.8%)	64 (88.9%)	0.004
Refer to Counsellor	58 (42.6%)			
Refer to Doctor	16 (11.8%)			
Refer to Social Worker	23 (16.9%)			
Refer to Specialist	19 (14.0%)			

* Perfect referrals = 100% referral of those diagnosed; imperfect referral = 0 diagnosed or < = 100% referral or diagnosed

MICA: Mental Illness Clinicians Attitude Scale; ACCEPTABILITY: Perceptions of Innovation Acceptability (screening, assessing, treating, referring); ORIC: Organizational Readiness for Implementing Change; APC: Adult Primary Care; BMH: Brief Mental Health Screening Tool

**Table 6:** Stage two cross-sectional provider survey participant characteristics and associations with diagnosis and referral: Logistic regression models (n = 136).

Variables	Coefficient	Std Err	t-value	p-value	95% CI
Married	0.600	0.27	-1.14	0.256	[0.249–1.448]
Facility Manager	0.252	0.138	-2.51	0.012	[0.086–0.738]
Other	0.787	0.922	-0.20	0.838	[0.079–7.819]
Registered Nurses					
Overtime	0.861	0.442	-0.29	0.770	[0.315–2.353]
Nightshift	0.240	0.135	-2.53	0.011	[0.080–0.725]
MICA	0.991	0.017	-0.57	0.570	[0.959–1.024]
ACCEPTABILITY	2.569	1.196	2.03	0.043	[1.031–6.400]
BMH TRAINING	3.747	2.194	2.26	0.024	[1.190–11.803]
Constant	0.021	0.043	-1.87	0.061	[0.000–1.204]
Observations	131				

* * *p <* .*05*

MICA: Mental Illness Clinicians Attitude Scale; ACCEPTABILITY: Perceptions of Innovation Acceptability (screening, assessing, treating, referring); BMH: Brief Mental Health Screening Tool

### Implementation

Two-thirds (68.4%) of cohort clinics had a clinic counsellor consistently available over the cohort period. Consistent availability of a clinic counsellor was associated with a greater probability of being referred (OR 5.9, 95%CI [1.29–27.75], *p* = 0.022) (See Table 4).

### Maintenance

Organizationally, several elements of the package have been adopted for scale up by the DOH in South Africa. The facility-based psychoeducational material was scaled up to all districts in KZN province by the KZN DoH during the COVID 19 pandemic (see www.crh.ukzn.ac.za). The KZN DoH also accepted the BMH screening tool as the standardized mental health screening tool to be used in PHC facilities, and training was conducted with the district mental health coordinators and district trainers to implement and train facility staff in the tool across the province (www.crh.ukzn.ac.za). The APC integrated clinical decision support tool and self-directed online 30 session APC training with additional mental health modules is available nationally (https://knowledgehub.health.gov.za/course/adult-primary-care-2020-update), together with the Wellness Resource, although the latter has not been formally adopted by the DoH. The task sharing of the facility-based counselling service has also not been formally adopted as of now.

## Discussion

Using RE-AIM as a guiding framework, this study has yielded several important lessons for optimizing implementation and uptake of integrated depression care within routine PHC clinic services. Regarding **Reach** at the PHC facility level, there was no significant change across the two stages in the percentage of PHC service users identified as having depression (roughly half) by nurse providers of those who screened above the established cut-off of ≤9 on the PHQ-9. This finding aligns with international findings of accurate detection of depression at PHC level [40]. Service users with more severe symptoms, suicidal thoughts, and perceived stress were more likely to be diagnosed with depression across both stages. Given scarce mental health professionals, these findings suggest that nurses in both stages were proficient at identifying service users most in need of treatment, based on symptom severity. A key finding was that the probability of referral was substantially higher in the second stage (over 90%), compared to the first stage (37%).

Regarding **adoption,** findings from the linked cross-sectional nurse survey in the second stage indicated nurses who found the integrated package acceptable as well as those in receipt of outreach training and technical support in the validated and mandated BMH screening tool were more likely to make a diagnosis, with most diagnosed patient participants also referred. A recent review suggests uncertainty about whether screening for depressive symptoms improves mental health outcomes as a result of better identification and treatment of people with depression who would otherwise have gone unrecognised [41]. As an adjunct tool to APC diagnostic treatment algorithms in PHC in South Africa, our results suggest that training and support in the use of standardized validated screening tools helps to alert nurses of the possibility of depression and the need to assess for possible diagnosis.

In relation to **implementation**, counsellor availability and greater role consonance with their training to become social auxiliary workers also impacted on diagnosis and referral. The lay counselling service was more consistently available and acceptable within the facilities in the second stage. This was due to the transitioning of HIV counsellors to social auxiliary workers, with limited mental health services part of their scope of practice. This addressed the problem of mental health counselling not previously being part of the scope of work of HIV counsellors, with their scope of practice restricted to pre- and post-test HIV and adherence counselling [42]. While more available and accepted in the facilities, the counsellors were, however, not always immediately present to receive a referral on the day of the referral as they were completing their training to become social auxiliary workers. This may have accounted for the lack of improvement in counselling uptake of referrals between the two stages. Referral for counselling was shown to be beneficial for service users with depression in the first stage evaluation [24], and thus a viable alternative to specialist counselling services in resource scarce settings.

Previous research suggests immediate linkage to counselling is important to promote counselling uptake [43]. Contextual factors may also have compounded service users’ capacity to attend clinics for counselling sessions. Off-site collection/distribution of medication was significantly expanded during the COVID 19 pandemic [44], and has since been maintained, reducing the number of PHC clinic visits by service users. The need for strengthening of community level mental health services is highlighted. To this end, through a SMhINT supplementary grant, a Community Mental Health Education and Detection (CMED) tool was co-created with the KZN DoH following the second stage. This tool uses vignettes and a prototype matching approach to assist community health works (CHWs) to provide mental health education, screening and linkage to care in their routine household visits [45–47]. The need for future research to evaluate its role in increasing demand and uptake of mental health and social services is indicated. CHWs are well placed to link community members to both health and social interventions where indicated; this being important given the well-established social determinants of poor mental health [38, 39].

In pursuit of narrowing the care gap for depression, the importance of whole systems strengthening along the PHC care cascade is underscored. While the percentage of screen positive service user participants who received the co-located counselling service was still very low in the second stage (8% of the original sample who screened positive for depressive symptoms using the PHQ9), it was far higher than the percentage of service users suspected of having a CMD based on national prevalence rates [48] initiated on treatment by limited PHC doctors/specialists using routine data for the district, estimated to be 0.19% [49]. Given that the efficacy of lay counselling services under controlled trial conditions is now well-established in LMICs, including South Africa [19, 30, 50], our study highlights the potential of task sharing of co-located counselling services in PHC for helping to narrow the care gap for depression, estimated to be as high as 97% in LMICs [2]. The need to mandate a health worker cadre to provide this service under supervision has been previously highlighted [43]. Further, given the shortage of primary health care doctors outside of the metropolitan areas of South Africa [51], the need for legislative and regulatory changes to enable nurses, who form the backbone of the PHC system in South Africa, to initiate antidepressant medication as contained in the new South African Mental Health Policy Framework and Acton Plan (2023–2030) [52], is underlined.

Regarding **maintenance**, the establishment of a learning collaborative with the researchers and provincial policy makers provided the platform for facilitating the adoption of the majority of the tailored implementation strategies into policy. This was central to strengthening the acceptability of the implementation package by the providers in the district, as well as creating an enabling policy environment for sustainability and scale-up of components of the implementation package to the rest of the province of KZN. To this end, the learning collaborative was expanded during the COVID-19 pandemic to include all the mental health coordinators across all 11 districts in the KZN province. Working incrementally and starting with screening, using routine data and continuous quality improvement small tests of change, SMhINT has supported the KZN DoH to introduce and embed the BMH into all districts, with a view to using the same approach for the other tools along the cascade of care. The work of SMhINT has also been integrated into a broader initiative to strengthen comprehensive integrated primary health care in the KZN province to facilitate maintenance and scale up.

### Limitations

The lack of engagement of people with lived experience within the learning collaborative and the focus on inputs and outcomes without also looking at quality of services from service user perspectives are recognized. The nurse diagnosis and referral checklist may have primed nurses to make a diagnosis thus inflating the percentage of service user participants who screened positive for depressive symptoms who were diagnosed. The observational design poses a limitation for evaluation of effectiveness and causal inference. A randomized control trial design was precluded by the adoption of a learning collaborative strategy necessary to create an enabling policy environment, with policy changes applied to all districts across the province, including the target district where the research was conducted. The restriction of focus on the PHC clinic facility level of care (with little attention to community care) was also a limitation. Although, as indicated, the CMED has since been developed using a SMhINT supplementary grant to provide an educational tool for CHWs to increase demand for services through psychoeducation, screening and linkage to care at a community level.

### Conclusion

Integrated depression care within routine PHC clinic services can be successfully implemented in resource-constrained settings through integrating depression services into routine activities for other medical conditions along the care cascade. Establishing a learning collaborative with policy makers, researchers and implementers is key to creating an enabling policy environment that ensures that successful strategies are mandated, including tools and revisions to health care worker roles and responsibilities. A validated, mandated mental health screening tool, embedded in the system using on-site educational outreach and technical support visits together with small tests of change, emerged as important for assisting Professional Nurses to diagnosis and refer service users with depression in this resource-constrained real-world PHC service delivery context. Further research efforts are, however, required to narrow the treatment uptake gap and assess quality of care. To this end, research is needed to assess whether improved availability of on-site treatment for depression as well as improved population mental health literacy may assist to improve treatment uptake and quality. While adequate mental specialist resources will always be necessary for training, supervision and providing referral resources for complex severe mental health conditions, the notion that LMICs potentially have a wealth of human resources to draw on to narrow the treatment gap for common mental conditions is supported [53].

## Supporting information

S1 Checklist STROBE statement—Checklist of items that should be included in reports of observational studies. (DOCX)

S1 Appendix TIDieR framework for the psychoeducational materials. (DOCX)

S2 Appendix TIDieR framework for mental health screening at PHC–Brief Mental Health (BMH) screening tool. (DOCX)

S3 Appendix TIDieR framework for the APC full course online. (DOCX)

S4 Appendix TIDieR framework for the APC wellness resource. (DOCX)

S5 Appendix TIDieR framework for the SMhINT counselling intervention. (DOCX)

S1 Text Inclusivity in global research. (DOCX)

## References

[1] Hajat C, Stein E. The global burden of multiple chronic conditions: A narrative review. Prev Med Rep. 2018;12:284–93. Epub 2018/11/09. doi: 10.1016/j.pmedr.2018.10.008 ; PubMed Central PMCID: PMC6214883. 30406006 PMC6214883

[2] Moitra M, Santomauro D, Collins PY, Vos T, Whiteford H, Saxena S, et al. The global gap in treatment coverage for major depressive disorder in 84 countries from 2000–2019: A systematic review and Bayesian meta-regression analysis. PLoS medicine. 2022;19(2):e1003901. Epub 2022/02/16. doi: 10.1371/journal.pmed.1003901 ; PubMed Central PMCID: PMC8846511. 35167593 PMC8846511

[3] Collaborators GBDMD. Global, regional, and national burden of 12 mental disorders in 204 countries and territories, 1990–2019: a systematic analysis for the Global Burden of Disease Study 2019. Lancet Psychiatry. 2022;9(2):137–50. Epub 2022/01/14. doi: 10.1016/S2215-0366(21)00395-3 ; PubMed Central PMCID: PMC8776563. 35026139 PMC8776563

[4] Moussavi S, Chatterji S, Verdes E, Tandon A, Patel V, Ustun B. Depression, chronic diseases, and decrements in health: results from the World Health Surveys. Lancet. 2007;370(9590):851–8. doi: 10.1016/S0140-6736(07)61415-9 . 17826170

[5] Arokiasamy P, Uttamacharya U, Jain K, Biritwum RB, Yawson AE, Wu F, et al. The impact of multimorbidity on adult physical and mental health in low- and middle-income countries: what does the study on global ageing and adult health (SAGE) reveal? BMC medicine. 2015;13:178. Epub 2015/08/05. doi: 10.1186/s12916-015-0402-8 ; PubMed Central PMCID: PMC4524360. 26239481 PMC4524360

[6] Ivbijaro G, Kolkiewicz L, Lionis C, Svab I, Cohen A, Sartorius N. Primary care mental health and Alma-Ata: from evidence to action. Mental health in family medicine. 2008;5(2):67–9. Epub 2008/06/01. ; PubMed Central PMCID: PMC2777560. 22477849 PMC2777560

[7] Keynejad R, Spagnolo J, Thornicroft G. WHO mental health gap action programme (mhGAP) intervention guide: updated systematic review on evidence and impact. Evid Based Ment Health. 2021. Epub 2021/04/28. doi: 10.1136/ebmental-2021-300254 ; PubMed Central PMCID: PMC8311089. 33903119 PMC8311089

[8] Katon WJ, Lin EH, Von Korff M, Ciechanowski P, Ludman EJ, Young B, et al. Collaborative care for patients with depression and chronic illnesses. N Engl J Med. 2010;363(27):2611–20. Epub 2010/12/31. doi: 10.1056/NEJMoa1003955 ; PubMed Central PMCID: PMC3312811. 21190455 PMC3312811

[9] Araya R, Alvarado R, Minoletti A. Chile: an ongoing mental health revolution. Lancet. 2009;374(9690):597–8. doi: 10.1016/S0140-6736(09)61490-2 . 19699997

[10] Araya R, Rojas G, Fritsch R, Gaete J, Rojas M, Simon G, et al. Treating depression in primary care in low-income women in Santiago, Chile: a randomised controlled trial. Lancet. 2003;361(9362):995–1000. Epub 2003/03/28. doi: 10.1016/S0140-6736(03)12825-5 . 12660056

[11] van Ginneken N, Tharyan P, Lewin S, Rao GN, Meera SM, Pian J, et al. Non-specialist health worker interventions for the care of mental, neurological and substance-abuse disorders in low- and middle-income countries. Cochrane Database Syst Rev. 2013;(11):CD009149. doi: 10.1002/14651858.CD009149.pub2 . 24249541

[12] Cubillos L, Bartels SM, Torrey WC, Naslund J, Uribe-Restrepo JM, Gaviola C, et al. The effectiveness and cost-effectiveness of integrating mental health services in primary care in low- and middle-income countries: systematic review. BJPsych Bull. 2020:1–13. Epub 2020/04/24. doi: 10.1192/bjb.2020.35 . 32321610 PMC8058938

[13] Wagenaar BH, Hammett WH, Jackson C, Atkins DL, Belus JM, Kemp CG. Implementation outcomes and strategies for depression interventions in low- and middle-income countries: a systematic review. Global Mental Health. 2020;7:e7. Epub 2020/03/02. doi: 10.1017/gmh.2020.1 32346482 PMC7176918

[14] Roomaney RA, van Wyk B, Cois A, Pillay-van Wyk V. One in five South Africans are multimorbid: An analysis of the 2016 demographic and health survey. PLoS One. 2022;17(5):e0269081. Epub 2022/05/27. doi: 10.1371/journal.pone.0269081 ; PubMed Central PMCID: PMC9135225. 35617298 PMC9135225

[15] Docrat S, Besada D, Cleary S, Daviaud E, Lund C. Mental health system costs, resources and constraints in South Africa: a national survey. Health Policy Plan. 2019;34(9):706–19. Epub 2019/09/24. doi: 10.1093/heapol/czz085 ; PubMed Central PMCID: PMC6880339. 31544948 PMC6880339

[16] Mash B, Fairall L, Adejayan O, Ikpefan O, Kumari J, Mathee S, et al. A morbidity survey of South African primary care. PLoS One. 2012;7(3):e32358. Epub 2012/03/24. doi: 10.1371/journal.pone.0032358 ; PubMed Central PMCID: PMC3306367. 22442666 PMC3306367

[17] Department of Health. Mental Health Policy Framework and Strategic Plan. Pretoria: Department of Health, 2013.

[18] Mahomed OH, Asmall S. Development and implementation of an integrated chronic disease model in South Africa: lessons in the management of change through improving the quality of clinical practice. International journal of integrated care. 2015;15:e038. Epub 2015/11/04. doi: 10.5334/ijic.1454 ; PubMed Central PMCID: PMC4628546. 26528101 PMC4628546

[19] Petersen I, Fairall L, Zani B, Bhana A, Lombard C, Folb N, et al. Effectiveness of a task-sharing collaborative care model for identification and management of depressive symptoms in patients with hypertension attending public sector primary care clinics in South Africa: pragmatic parallel cluster randomised controlled trial. J Affect Disord. 2021;282:112–21. Epub 2021/01/08. doi: 10.1016/j.jad.2020.12.123 . 33412490

[20] Hwang S, Birken SA, Melvin CL, Rohweder CL, Smith JD. Designs and methods for implementation research: Advancing the mission of the CTSA program. J Clin Transl Sci. 2020;4(3):159–67. Epub 2020/07/23. doi: 10.1017/cts.2020.16 ; PubMed Central PMCID: PMC7348037. 32695483 PMC7348037

[21] Petersen I, Kemp CG, Rao D, Wagenaar BH, Sherr K, Grant M, et al. Implementation and Scale-Up of Integrated Depression Care in South Africa: An Observational Implementation Research Protocol. Psychiatr Serv. 2021:appips202000014. Epub 2021/03/12. doi: 10.1176/appi.ps.202000014 . 33691487 PMC8410621

[22] Glasgow RE, McKay HG, Piette JD, Reynolds KD. The RE_AIM framework for evaluating interventions: what can it tell us about approaches to chronic illness managment? Patient Education and Counselling. 2000:44, 119–27. 10.1016/s0738-3991(00)00186-5 11479052

[23] Sweet SN, Ginis KAM, Estabrooks PA, Latimer-Cheung AE. Operationalizing the RE-AIM framework to evaluate the impact of multi-sector partnerships. Implementation Science 2014:9(74), 1–10. http://www.implementationscience.com/content/9/1/74. doi: 10.1186/1748-5908-9-74 24923331 PMC4072487

[24] Kathree T, Bachmann M, Bhana A, Grant M, Mntambo N, Gigaba S, et al. Management of Depression in Chronic Care Patients Using a Task-Sharing Approach in a Real-World Primary Health Care Setting in South Africa: Outcomes of a Cohort Study. Community Ment Health J. 2023. Epub 2023/03/26. doi: 10.1007/s10597-023-01108-y . 36964282 PMC10447595

[25] Bhana A, Rathod SD, Selohilwe O, Kathree T, Petersen I. The validity of the Patient Health Questionnaire for screening depression in chronic care patients in primary health care in South Africa. BMC Psychiatry. 2015;15(1):118. doi: 10.1186/s12888-015-0503-0 ; PubMed Central PMCID: PMC4446842. 26001915 PMC4446842

[26] Kemp CG, Mntambo N, Weiner BJ, Grant M, Rao D, Bhana A, et al. Pushing the bench: A mixed methods study of barriers to and facilitators of identification and referral into depression care by professional nurses in KwaZulu-Natal, South Africa. SSM Ment Health. 2021;1. Epub 2021/09/21. doi: 10.1016/j.ssmmh.2021.100009 ; PubMed Central PMCID: PMC8443051. 34541564 PMC8443051

[27] Janse van Rensburg A, Kathree T, Breuer E, Selohilwe O, Mntambo N, Petrus R, et al. Fuzzy-set qualitative comparative analysis of implementation outcomes in an integrated mental healthcare trial in South Africa. Global health action. 2021;14(1):1940761. Epub 2021/08/18. doi: 10.1080/16549716.2021.1940761 ; PubMed Central PMCID: PMC8381905. 34402770 PMC8381905

[28] Powell BJ, Waltz TJ, Chinman MJ, Damschroder LJ, Smith JL, Matthieu MM, et al. A refined compilation of implementation strategies: results from the Expert Recommendations for Implementing Change (ERIC) project. Implementation science: IS. 2015;10:21. Epub 2015/04/19. doi: 10.1186/s13012-015-0209-1 ; PubMed Central PMCID: PMC4328074. 25889199 PMC4328074

[29] Fairall L, Bateman E, Cornick R, Faris G, Timmerman V, Folb N, et al. Innovating to improve primary care in less developed countries: towards a global model. BMJ Innov. 2015;1(4):196–203. doi: 10.1136/bmjinnov-2015-000045 ; PubMed Central PMCID: PMC4680195. 26692199 PMC4680195

[30] Singla DR, Kohrt BA, Murray LK, Anand A, Chorpita BF, Patel V. Psychological Treatments for the World: Lessons from Low- and Middle-Income Countries. Annual review of clinical psychology. 2017;13:149–81. doi: 10.1146/annurev-clinpsy-032816-045217 ; PubMed Central PMCID: PMC5506549. 28482687 PMC5506549

[31] Petersen I, Selohilwe O, Georgeu-Pepper D, Ras CJ, Zani B, Petrus R, et al. A collaborative care package for depression comorbid with chronic physical conditions in South Africa. BMC health services research. 2022;22(1):1465. Epub 2022/12/03. doi: 10.1186/s12913-022-08874-7 ; PubMed Central PMCID: PMC9717432. 36457094 PMC9717432

[32] Kemp CG, Mntambo N, Bachmann M, Bhana A, Rao D, Grant M, et al. Patient-level predictors of detection of depressive symptoms, referral, and uptake of depression counseling among chronic care patients in KwaZulu-Natal, South Africa. Glob Ment Health (Camb). 2020;7:e18. Epub 2020/09/12. doi: 10.1017/gmh.2020.11 ; PubMed Central PMCID: PMC7443607. 32913657 PMC7443607

[33] Petersen I, Bhana A, Fairall LR, Selohilwe O, Kathree T, Baron EC, et al. Evaluation of a collaborative care model for integrated primary care of common mental disorders comorbid with chronic conditions in South Africa. BMC Psychiatry. 2019;19(1):107. Epub 2019/04/05. doi: 10.1186/s12888-019-2081-z ; PubMed Central PMCID: PMC6448306. 30943947 PMC6448306

[34] Whiteford HA, Harris MG, McKeon G, Baxter A, Pennell C, Barendregt JJ, et al. Estimating remission from untreated major depression: a systematic review and meta-analysis. Psychological medicine. 2013;43(8):1569–85. Epub 2012/08/14. doi: 10.1017/S0033291712001717 . 22883473

[35] Cotterill S, Knowles S, Martindale AM, Elvey R, Howard S, Coupe N, et al. Getting messier with TIDieR: embracing context and complexity in intervention reporting. BMC medical research methodology. 2018;18(1):12. Epub 2018/01/20. doi: 10.1186/s12874-017-0461-y ; PubMed Central PMCID: PMC5774137. 29347910 PMC5774137

[36] Rudd BN, Davis M, Beidas RS. Integrating implementation science in clinical research to maximize public health impact: a call for the reporting and alignment of implementation strategy use with implementation outcomes in clinical research. Implementation science: IS. 2020;15(1):103. Epub 2020/11/27. doi: 10.1186/s13012-020-01060-5 ; PubMed Central PMCID: PMC7690013. 33239097 PMC7690013

[37] Proctor EK, Powell BJ, McMillen JC. Implementation strategies: recommendations for specifying and reporting. Implementation science: IS. 2013;8:139. Epub 2013/12/03. doi: 10.1186/1748-5908-8-139 ; PubMed Central PMCID: PMC3882890. 24289295 PMC3882890

[38] Bhana A, Mntambo N, Gigaba SG, Luvuno ZPB, Grant M, Ackerman D, et al. Validation of a brief mental health screening tool for common mental disorders in primary healthcare. S Afr Med J. 2019;109(4):278–83. Epub 2019/05/16. doi: 10.7196/SAMJ.2019.v109i4.13664 . 31084695 PMC7029170

[39] Curran R, Murdoch J, van Rensburg AJ, Bachmann M, Awotiwon A, Ras CJ, et al. A health systems intervention to strengthen the integration of tuberculosis and COVID-19 detection: Outcomes of a quasi-experimental study in a high burden tuberculosis district in KwaZulu Natal, South Africa. Trop Med Int Health. 2023. Epub 2023/02/09. doi: 10.1111/tmi.13860 . 36751975

[40] Mitchell AJ, Vaze A, Rao S. Clinical diagnosis of depression in primary care: a meta-analysis. Lancet. 2009;374(9690):609–19. Epub 2009/07/31. doi: 10.1016/S0140-6736(09)60879-5 . 19640579

[41] Thombs BD, Markham S, Rice DB, Ziegelstein RC. Does depression screening in primary care improve mental health outcomes? BMJ. 2021;374:n1661. Epub 2021/07/21. doi: 10.1136/bmj.n1661 . 34281908

[42] Petersen I, Fairall L, Egbe CO, Bhana A. Optimizing lay counsellor services for chronic care in South Africa: a qualitative systematic review. Patient Educ Couns. 2014;95(2):201–10. Epub 2014/03/19. doi: 10.1016/j.pec.2014.02.001 . 24629835

[43] Selohilwe O, Fairall L, Bhana A, Kathree T, Zani B, Folb N, et al. Challenges and opportunities for implementation and dissemination of a task- sharing counselling intervention for depression at primary health care level in South Africa. Int J Ment Health Syst. 2023;17(1):7. Epub 2023/03/31. doi: 10.1186/s13033-023-00575-w ; PubMed Central PMCID: PMC10064738. 36998053 PMC10064738

[44] Pillay Y, Museriri H, Barron P, Zondi T. Recovering from COVID lockdowns: Routine public sector PHC services in South Africa, 2019–2021. S Afr Med J. 2022;113(1):17–23. Epub 2022/12/21. doi: 10.7196/SAMJ.2022.v113i1.16619 . 36537548

[45] Grant M, Bhana A., Kathree T., Khuzwayo N., Van Rensburg A.J., Mthethwa L., et al. The feasibility of a Community Mental Health Education and Detection (CMED) Tool in South Africa. Social Science & Medicine Mental Health. 2023. 10.1016/j.ssmmh.2023.100188 PMC11189615 38903557

[46] Grant M, Luvuno Z, Bhana A, Mntambo N, Gigaba S, Ntswe E, et al. The development of a Community Mental Health Education and Detection (CMED) tool in South Africa. SSM-Mental Health. 2021;1:100023. doi: 10.1016/j.ssmmh.2021.100023 37274432 PMC10238082

[47] Grant M, Petersen I, Mthethwa L, Luvuno Z, Bhana A. Accuracy of a community mental health education and detection (CMED) tool for common mental disorders in KwaZulu-Natal, South Africa. Int J Ment Health Syst. 2022;16(1):44. Epub 2022/08/24. doi: 10.1186/s13033-022-00554-7 ; PubMed Central PMCID: PMC9400279. 35999643 PMC9400279

[48] Herman AA, Stein DJ, Seedat S, Heeringa SG, Moomal H, Williams DR. The South African Stress and Health (SASH) study: 12-month and lifetime prevalence of common mental disorders. S Afr Med J. 2009;99 (5 Pt 2):339–44. Epub 2009/07/11. ; PubMed Central PMCID: PMC3191537. 19588796 PMC3191537

[49] Department of Health SA. District Health Information System (DHIS2). 2022.

[50] MyersB, LombardCJ, LundC, JoskaJA, LevittN, NalediT, et al. Comparing dedicated and designated approaches to integrating task-shared psychological interventions into chronic disease care in South Africa: a three-arm, cluster randomised, multicentre, open-label trial. Lancet. 2022;400(10360):1321–33. Epub 2022/10/17. doi: 10.1016/S0140-6736(22)01641-5 .36244383

[51] Cooke R, Couper I, Versteeg M. Human resources for rural health. In: Padarath A, English R, editors. South African Health Review 2011. Durban: Health Systems Trust; 2011. p. 107–18.

[52] (NDoH). NDoH. National Mental Health Policy Framework and Strategic Plan 2023. Pretoria: National Department of Health, South Africa., 2023.

[53] Ndetei DM, Mutiso V, Osborn T. Moving away from the scarcity fallacy: three strategies to reduce the mental health treatment gap in LMICs. World Psychiatry. 2023;22(1):163–4. Epub 2023/01/15. doi: 10.1002/wps.21054 . 36640407 PMC9840495

