# Supplementary material for: Strengthening integrated depression services within routine primary health care using the RE-AIM framework in South Africa
Source: PLOS Glob Public Health. 2023 Nov 13;3(11):e0002604. doi: 10.1371/journal.pgph.0002604 (PMC10642780; doi:10.1371/journal.pgph.0002604)
Supplement: S1 Appendix — (DOCX) [file pgph.0002604.s002.docx]

**S1 Appendix: TIDieR framework for the Psychoeducational Materials**

| **1. Brief Name** | **Psychoeducational Materials** |
| --- | --- |
| **2. Why** | The initial psychoeducational package consisted of mental health and well-being waiting room educational materials including waiting room talks on depressive symptoms and where to get help, with an adjunct educational leaflet. In the context of the negative impact of COVID 19 on people’s mental health, the need to expand on the existing educational material for primary health care (PHC) clinics to include additional concerns related to increased anxiety, grief and bereavement and medication adherence as well as self-help strategies was highlighted. |
| **3. What materials** | **Expanded educational materials for service users:** The expanded educational material consisted of waiting room talks that covered four topics - depression, anxiety, loss and grief and adherence to chronic medication. Vignettes of a person having the symptoms of the condition/struggling with the issue were used to introduce a facilitated discussion on the symptoms/problems the person in the vignette was experiencing to raise awareness of the condition/problem.  **Adjunct educational leaflets/posters for service users:** Each topic had an adjunct poster/pamphlet, that used the same vignette to introduce the condition/problem. Information on what to do to deal with the symptoms of the condition/problem covered self-help lifestyle information, digitalized self-help skills building educational material, as well as where get telephonic and face to face treatment.  **Educational materials for providers**: A power point presentation was developed to assist mental health coordinators to educate service providers in the delivery of the waiting room talks. |
| **4. What procedures** | A train the trainer strategy was used to cascade the training down to the provider in the PHC clinics responsible for routine waiting room morning talks |
| **5a. Who provided** | A project employed Adult Education Trainer serviced as the master trainer and provided the digitalized educational meetings with the project employed Registered Psychological Counsellor/Supervisor who was responsible for training and supervision of the clinic HIV counsellors who provided the morning talks. |
| **5b. Who received** | District mental health coordinator and clinic counsellors |
| **5c. Who benefits** | - HIV counsellors who were trained to use the psychoeducational material were capacitated to deliver psychoeducation to all PHC clinic service users - Service users in the waiting rooms and those receiving mental health (depression and anxiety) counselling |
| **6. How** | - Education of the Registered Psychological Counsellor/Supervisor was provided online by the adult education specialist. Training of the clinic HIV counsellors was provided through face-to-face educational meetings in the district, followed by outreach on-site education as well as telephonic technical support as required. |
| **7. Where** | Educational meetings were held at the District office |
| **8. When and how much** | Three- day educational meetings at District office with individual in-vivo sessions with each HIV counsellor at their respective facilities followed by telephonic and on-site technical support as required. |
| **9. Tailoring** | Materials and associated training were tailored to include the impact of COVID-19 on mental health and wellbeing during the pandemic. |
| **10. Modifications** | Reference to COVID-19 has since been removed from the current posters/pamphlets |
| **11. How well-planned?** | Continuous Quality Improvement (CQI) was planned to support the implementation of the strengthened psychoeducational package in Amajuba through audit and feedback and small tests of change. |
| **12. How well-actual** | The CQI mentor audited and provided feedback to the Operational Manager of each clinic to ensure that materials were available as required, namely, that the psychoeducational posters were displayed in all the waiting rooms in each facility, and sufficient waiting room talks manuals, psychoeducational pamphlets were available at each facility. The Registered Psychological Counsellor/Supervisor provided telephonic and on-site support to the counsellors using the materials. |
