# Supplementary material for: Strengthening integrated depression services within routine primary health care using the RE-AIM framework in South Africa
Source: PLOS Glob Public Health. 2023 Nov 13;3(11):e0002604. doi: 10.1371/journal.pgph.0002604 (PMC10642780; doi:10.1371/journal.pgph.0002604)
Supplement: S2 Appendix — (DOCX) [file pgph.0002604.s003.docx]

**S1 Appendix: TIDieR Framework for Mental Health Screening at PHC - Brief Mental Health Screening (BMH)**

| **1. Brief name** | **Strengthening Mental Health Screening Services at PHC** |
| --- | --- |
| **2. Why** | The need for strengthening the mental health screening process was identified in first phase of the SMhINT evaluation which revealed the lack of a validated mental health screening tool within PHC clinics. Screening processes were also unstandardized and unsystematic, with professional nurses (PNs) not trusting the screening process. The goal of the intervention was thus to introduce a validated mandated screening tool, together with standardizing the administration processes thereof. |
| **3. What materials** | **The Brief Mental Health screening tool (BMH):**  The Brief Mental Health (BMH) Screening Tool was validated by the SMhINT research team (<http://www.samj.org.za/index.php/samj/article/view/12579>). It screens for common mental disorders (CMDs) (Depression, Anxiety and Harmful Alcohol use) in adults and was translated into isiZulu. The tool consists of a four-point Likert-scale allowing patients to indicate whether they have the symptom/s of each of the screened CMDs and the frequency with which they experienced the symptom/s. Based on the outcome, a patient is identified as needing further mental health assessment during the clinical consultation or advised to receive their routine care without further mental health intervention. The tool consists of a scoring guide as well as a chart for Standard Alcohol drinks to assist patients in identifying the type and units of alcohol they consume.  **The BMH Poster**: A poster version of the BMH that could be displayed in the screening areas for increased visibility  **The BMH Standard Operating Procedure (SoP):** A Department of Health (DoH) approved SoP, providing supporting educational material detailing the process of administration, scoring and recording screening outcomes, as well as outlining referral pathways for patients with significant outcomes or who require emergency care.  **The BMH Training Guide:** Educational material on how to administer and score the BMH, including a power point presentation template for district and facility-based trainers to use when training their facilities |
| **4. What procedures** | A train the trainer strategy was used to cascade the training down to the provider in the PHC clinics responsible for mental health screening. |
| **5a. Who provided** | A project employed Adult Education Trainer serviced as the master trainer and provided the digitalized educational meetings with the project employed Implementation Coordinator and Department of Health employed Mental Health Coordinator responsible for mental health services in the district. Both then provided training and CQI support supervision of the clinic HIV counsellors who provided the morning talks. |
| **5.b. Who received** | District Mental Health Coordinator  Project employed Implementation Coordinator  Enrolled Nurses (N=34) who provided other routine screening services in PHC clinics  Professional Nurses (N=126) who provide clinical services in PHC clinics  Operational Managers were also trained in order to provide support for implementation of mental health screening (N=19). |
| **5.c. Who benefits** | Enrolled nurses who are capacitated with a validated screening tool for service users  Professional Nurses who have a validated screening tool to screen service users, and who are prompted by a trustworthy screening tool to assess service users for possible depression, anxiety and alcohol misuse.  Service users at risk of depression and anxiety and alcohol misuse who are more likely to be referred, assessed, diagnosed and treated |
| **6. How** | Education of the Implementation coordinator and Mental Health Coordinator was provided online by the adult education specialist. Educational outreach visits to each PHC clinic provided face-to-face training, mentorship and continuous quality improvement support in using the tool through small tests of change. |
| **7. Where** | Online educational outreach via the Zoom platform  Vital signs station and consultation rooms in PHC clinics |
| **8. When and how much?** | Educational outreach visits at the clinics lasted between one to three hours, depending on whether one-one or group sessions were conducted. |
| **9. Tailoring** | Initial tailoring involved fixing a large laminated copy of the BMH tool on the ENAs’ desks for easy access to the BMH questions whilst conducting screening.  PHC clinics adapted provision of mental health screening to suit their context i.e. some screening is done in vital signs station by ENs and in consulting rooms by PNs in other PHC clinics. |
| **10. Modifications** | No modifications to the materials or the educational outreach |
| **11. How well planned** | All ENs and PNs in 19 PHC clinics were meant to receive the face-to-face educational outreach visits. |
| **12. How well actual** | All 19 facilities received educational outreach and follow-up support from the CQI mentor. |
