# Supplementary material for: Strengthening integrated depression services within routine primary health care using the RE-AIM framework in South Africa
Source: PLOS Glob Public Health. 2023 Nov 13;3(11):e0002604. doi: 10.1371/journal.pgph.0002604 (PMC10642780; doi:10.1371/journal.pgph.0002604)
Supplement: S3 Appendix — (DOCX) [file pgph.0002604.s004.docx]

**S3 Appendix: TIDieR framework for the APC Full course online**

| **1. Brief name** | **Adult Primary Care (APC) full course online** |
| --- | --- |
| **2. Why** | The Adult Primary Care Clinical Support Tool is symptom-based and integrated, using algorithms and checklists to guide the health worker to assess, advise and treat the patient’s condition/s.  Concise and simple to use, APC is also comprehensive, covering the identification and management of more than 80 common symptom presentations and over 30 chronic conditions, including non-communicable diseases, communicable diseases, mental health conditions, women’s health and palliative care.  The APC full course online was developed in response to the COVID-19 pandemic, where lockdown stopped the face-to-face Educational meetings option due to infection control safety. There was still a need for APC educational meetings in the services, so the content was transferred to the KTU online school.  Within the SMhINT programme in Amajuba, getting nurses to do online educational meetings that is not part of the Ideal Clinic Initiative was challenging, so support to do the APC full course, which covers four Mental Health cases was seen as the most viable option of leading nurses to the APC mental health content. |
| **3. What materials** | The APC Full course online content is the same as the paper-based 2020 version of the educational materials, but instead of the cases needing to be facilitated, users work through cases structured as a quiz online. The course content is scaffolded to start with simple cases that ensure the users learn about the features of APC. The cases become more complicated, to ensure users learn how to navigate all sections of APC confidently. On completion a certificate is autogenerated.  The four mental health cases in APC cover the following learning objectives:   1. Sandile  - Navigating the Mental Health Care Act for an acutely psychotic patient  1. Jane  - Recognising, diagnosing and managing the patient with depression  1. Adelaide  - A symptom (dyspepsia) prompting the diagnosis of a chronic condition (alcohol misuse and harm) - To incorporate supporting the patient to change behaviour into a consultation  1. Sindi  - To increase confidence when managing a suicidal client. - Recognising and managing depression in a postnatal patient.   The APC Guide was provided to facilities during 2020 and 2021 which proved useful for staff to have hard copies of APC when doing the online course. |
| **4. What procedures** | The need to strengthen the mental health component of primary health care in the Amajuba District as part of the SMhINT programme necessitated the upskilling of nurses with the use of APC.  APC online champions were identified for each facility to be able to assist their colleagues to access the online APC Full Course. A facilitator online educational meeting was run in September 2021. Apart from assisting colleagues to access the online educational meetings, they were also mandated to support completion of the APC online educational meetings.  Progress reports were designed and generated 2 weekly from December 2021 to be able to send via a PDF on WhatsApp to each APC online champion so they could track process of completion in order to know where support was needed. Below is an image of one of these reports:  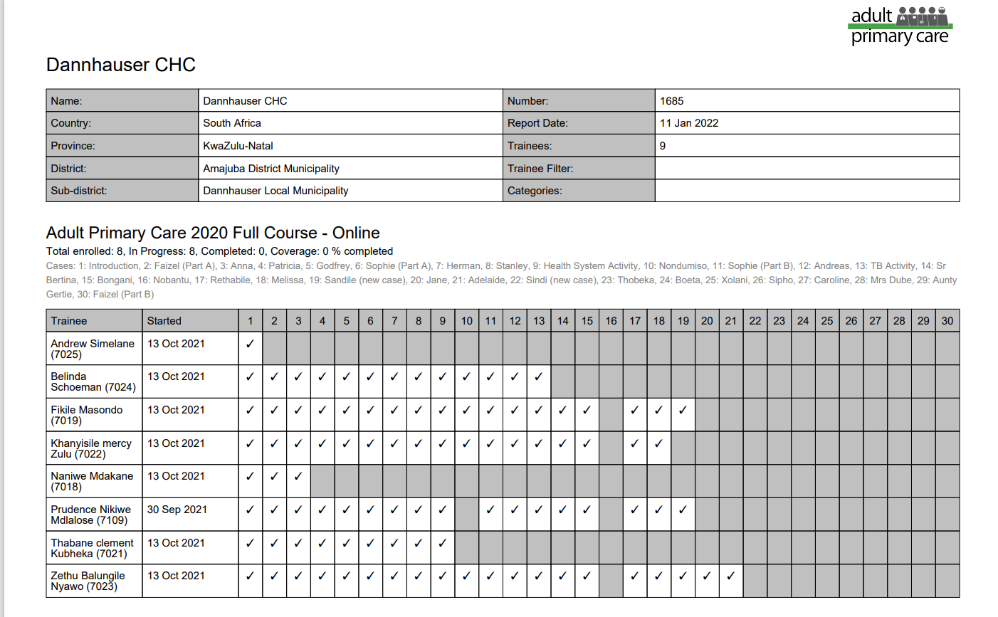 |
| **5a. Who provided** | KTU master trainers trained the Implementation co-ordinator and APC online champions in September 2021.  On the ground follow-up technical support was provided to each clinic APC online champion by the Implementation Coordinator. |
| **5b. Who received** | APC online champions were trained in all 24 PHC clinics that include the 19 PHC clinics where the implementation strategy was evaluated to support the nurses in the clinics to access the APC Full Course online. |
| **5c. Who benefits** | - Nurses who manage their patients correctly, according to guidelines and policy. - Patients who are diagnosed, treated, managed and referred correctly. - Doctors see correctly referred patients and less incorrectly referred patients. - The health system is strengthened by staff working according to policy recommendations that are up to date and evidence based. This aims to ensure best practice. |
| **6. How** | The access link was sent out on WhatsApp groups/email. The users are prompted to click on the link and create a user profile to access the course. |
| **7. Where** | - Self-directed, online (phone/laptop). - Small group sessions where nurses work through the online content on individual devices but together for discussion and shared learning.   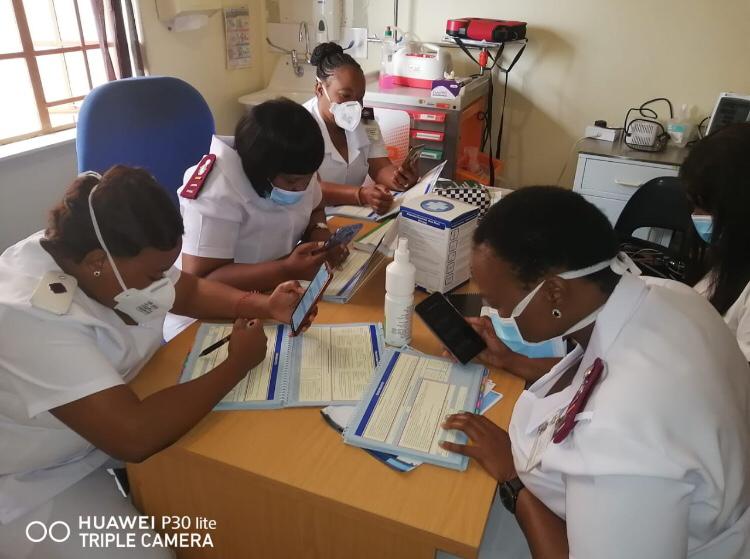   - Data free to the 4 major mobile networks (this means there is no cost to the user if they belong to Vodacom, MTN, Cell C or Telkom). |
| **8. When and how much** | - The APC full course has 27 cases and 3 activites in total. - It can be done at own pace. - Helpful to be done with others to support health systems strengthening as a team approach. - Certificate on completion of all content in the resource. |
| **9. Tailoring** | Initially in the SMhINT programme nurses were expected to complete a specific Mental Health APC Educational meetings module, however as this was not mandated by the District, and seen as extra work, the request was to change to the APC full course. |
| **10. Modifications** | No modifications have been made to the current edition of the APC full course. |
| **11. How well - planned** | After the educational meetings of APC facility champions the champions were mandated to give feedback to their facilities and to assist their teammates to enrol on the online course depending on their certification. They were also supported by the Amajuba District educational meetings coordinator, District Family Physician, Operational managers, and the CRH team. |
| **12. How well - actual** | Of a total of 269 nurses on the ground in Amajuba (information from baseline surveys) 161 (60%) have enrolled for APC Full course online. Of those enrolled 78 (48%) have completed the course. The CRH team sent reports via WhatsApp to each champion every 2 weeks and visited the facility at least once or twice a month. The support visit was for discussing progress, understanding the challenges, assisting with technical issues, such as passwords, lack of time due to work and staff shortages; and coming up with possible solutions. Issues such as a lack of time for online learning were discussed with district leads and there was an agreement that there should be allocated time weekly for staff to do online courses, not only APC, as there were other courses that had to be done online. This plan was implemented using CQI small tests of change to support and monitor if the plan works. Operational Managers were supportive of this plan and allocated time to staff who could do the course, based on the clinic flow. Each Professional nurse was supported by the champions based on their progress to move on to the next case. Where champions had challenges in supporting colleagues, they would ask the Operational Manager and Implementation Coordinator to assist with discussing possible solutions and prompting the team to use the online platform for learning. Facilities with high patient load as well as high staff turn-over were unable to progress at the same pace as facilities with a usual patient flow had to be given more time to progress. |
