# Supplementary material for: Strengthening integrated depression services within routine primary health care using the RE-AIM framework in South Africa
Source: PLOS Glob Public Health. 2023 Nov 13;3(11):e0002604. doi: 10.1371/journal.pgph.0002604 (PMC10642780; doi:10.1371/journal.pgph.0002604)
Supplement: S4 Appendix — (DOCX) [file pgph.0002604.s005.docx]

**S4: TIDieR framework for the APC Wellness Resource**

| **1. Brief name** | **APC Wellness Resource** |
| --- | --- |
| **2. Why** | The APC Wellness Resource was developed in response to the finding that for nurses to be able to diagnose and refer there was a need to care for their own emotional wellbeing. During the pandemic, frontline workers were also exposed to trauma brought about by the increased workload, rising death rate as well as overwhelming and distressing emotional events.  Based on the evidence from literature reviews on the impact of pandemics on mental health, the on-the-ground relationships of the research team as well as a request from the KwaZulu-Natal Mental health Directorate, the APC Wellness Resource was designed to offer frontline clinicians, and in particular nurses, self-care tools. This resource was designed to empower nurses to care for their mental health and promote resilience, especially during the pandemic.  Given nurses time constraints, and the existing stigma associated with mental health challenges, the APC Wellness Resource provides a self-directed, online resource that can be done at the end-users own pace and in their own time. |
| **3. What materials** | The APC Wellness Resource educational material follows the same approach as the APC guide that uses vignettes of characters in a clinic tea room who share their common mental health challenges.  These vignettes/characters integrate the following resources:   - APC guide (a clinical practice guide familiar to all nurses in PHC) - The psycho-educational materials, namely:   - Four pamphlets/posters   - Self-help skills videos that promote healthy thinking, problem management, containing leadership, dealing with loss and grief and how to give bad news   - A workbook that helps make the videos practical and allows the user to track their self-care, wellness journey. - An emotional health thermometer is also used as a gauge to check when and what help is required to revert to a healthier state of wellbeing. Disruption to our emotional health, causing our emotional health thermometers to rise to the red zone (overwhelmed), orange (anxious), green (in control). - Other resources such as breathing techniques and the Infection, Prevention and Control (IPC) guideline are included as needed.   The following infographic describes the characters, their issues and the information and skills learnt through using the APC Wellness Resource. Being a tea room scene, they find ways to share their stories with their colleagues, in the hope that the impact of mental health issues can be destigmatised and normalised to some extent.   |
| **4. What procedures** | - The APC Wellness Resource piggy-backed on the Knowledge Translation Unit’s (KTU’s) APC online resources using Thinkifik, an online training programme platform. - This platform provides access to who uses the resources, what modules they cover and any other feedback the end-user might provide. - The pilot testing phase took place during February 2021 and was launched in **May 2021** for access by all District clinics. - The APC Wellness Resource was also promoted by the KZN Mental Health Directorate during a mental health promotion month and the infographic above was used to promote the resource. |
| **5a. Who provided** | Project employed Implementation Coordinator |
| **5b. Who received** | Nurses in PHC clinics in the Amajuba District. |
| **5c. Who benefits** | - Nurses who gain peace of mind by being equipped to deal with the impact of the additional mental and emotional burden working during and beyond the pandemic. - Managers who gain information and confidence to support staff. |
| **6. How** | The APC Wellness Resource was launched at a District learning session in May 2021, where the Operational Managers attending were orientated to it and given access to the link for distribution to their staff. The access link was sent out on WhatsApp groups/email. The user was prompted to click on the link and create a user profile to access the resource. |
| **7. Where** | - Self-directed, online (Phone/laptop) - Data free to the 4 major mobile networks (this means there is no cost to the user if they belong to Vodacom, MTN, Cell C or Telkom) |
| **8. When and how much** | This resource is essentially for nurses who are familiar with APC but any health care provider will be able to benefit from this resource.   - Content not compulsory – choose topics relevant to their needs - Done at own pace - Helpful to be done with others - Certificate on completion of all content in the resource |
| **9. Tailoring** | **Pilot phase**  Purposive sampling of end-users from the Amajuba district including members of the KZN DoH Mental health Directorate and Mental health specialists were approached during the pilot phase in the development of this resource. Their recommendations included the following changes:   - To include a workbook to use alongside the resource - To include the emotional health thermometer |
| **10. Modifications** | - No modifications have been made to the current edition of this psycho-educational resource. |
| **11. How well - planned** | - The design of the resource was for people to decide what parts resonated with their need, there was no instruction or expectation that all content had to be completed. - This resource was created as a response to the need of nurses in crisis during the pandemic. It was planned to be open to anyone who would find it helpful. It was a self-directed resource, as there was no allowance for face-to-face support at the time it was launched. - This resource was offered free to mobile users from the 4 major cellular networks. It is not possible to track users who are not able to access the data free service. |
| **12. How well - actual** | As of 27 June 2022, there have been 63 enrolments into the APC Wellness Resource in Amajuba and 7 completions.   \| Of the 63 enrolments: \| Chapter name: \| \| --- \| --- \| \| 36 % of enrolments completed: \| Managing stress for a healthier lifestyle (this was a prerequisite chapter to continue with the rest of the content) \| \| 21 % of enrolments completed: \| Sr Buthelezi: problem management \| \| 16 % of enrolments completed: \| Sr Johnson: self-compassion when giving bad news \| \| 11% of enrolments completed: \| Mr Mthembu: coping with stress and anxiety \| \| 11% of enrolments completed: \| Sr February: identifying occupational stress and seeking professional help \| \| 11% of enrolments completed: \| Mr Ward: Coping with grief and loss \| \| 11% of enrolments completed: \| The whole resource \| |
