# Supplementary material for: Strengthening integrated depression services within routine primary health care using the RE-AIM framework in South Africa
Source: PLOS Glob Public Health. 2023 Nov 13;3(11):e0002604. doi: 10.1371/journal.pgph.0002604 (PMC10642780; doi:10.1371/journal.pgph.0002604)
Supplement: S5 Appendix — (DOCX) [file pgph.0002604.s006.docx]

**S5 Appendix: TIDieR framework for the SMhINT counselling intervention**

| **1. Brief name** | **Counselling intervention** |
| --- | --- |
| **2. Why** | The counselling intervention is based on adapted CBT techniques (problem management and healthy thinking), It was made up of 6 sessions and an additional adherence session called ‘Getting to know your chronic condition(s) and medication’. Five sessions were centred on triggers and issues that maintain depressive cycles (feeling anxious because of COVID-19; Worrying about making ends meet; feeling stigmatised and discriminated against; feeling overwhelmed by relationship problems and understanding grief and loss). The sixth session focused on depression psychoeducation. Each session was supported with corresponding self-help skills building videos and information pamphlet. |
| **3. What materials** | A train the trainer strategy was used to capacitate clinic counsellors in the provision of counselling to referred service-users. To this end, a suite of educational materials was designed to enable trainers/supervisors to capacitate the clinic counsellors in the counselling intervention, with clinic counsellors also provided with educational materials in the form of a manual and supporting material.  **Educational materials:**   1. Trainer- Supervisor manual - a step-by-step instructional educational tool to guide intervention trainers using detailed step-by-step instructions on how to train the Clinic Counsellors in the counsellor training programme. 2. Supervisor’s Guide to support supervisors to provide professional and regular supervision to promote competence and confidence in counsellors, and effectively manage the difficulties experienced with counselling. 3. Self-help videos using a stepped approach to self-care were used namely, healthy thinking skills, loss and grief and problem management skills were developed. These animated videos use English captions with isiZulu narration. 4. Information pamphlets and posters in English and isiZulu to be used as adjunct materials in the counselling sessions with links to the self-help skills building videos. 5. Chronic conditions educational pamphlets in English and isiZulu. Counselling intervention forms were included in the educational outreach sessions to ensure embedding use in practice |
| **4. What procedures** | HIV counsellors who were also selected by the District to be trained to be auxiliary social workers were given a group structured face-to-face educational meetings over three days. This was followed by *in vivo* apprenticeship training where the supervisor sat through three sessions facilitated by the counsellor and provided feedback on the counsellor’s counselling skills. The counsellors received onsite and telephonic individual clinical supervision where the supervisor helped with development of skills and provided emotional support. |
| **5a. Who provided** | Project employed Registered Psychological Counsellor provided the training via face to face educational meetings as well as apprenticeship training and supervision of the clinic counsellors. |
| **5b. Who received** | Twenty HIV lay-counsellors selected from 19 PHCs who were concurrently receiving educational outreach to be auxiliary social workers |
| **5c. Who benefits** | - HIV counsellors were capacitated to provide depression and anxiety counselling, as well as adherence counselling - Service users referred for the co-located counselling by the Professional Nurses who took up the counselling |
| **6. How** | HIV counsellors were provided with three-day face-to-face training educational workshops. This was followed by *in vivo* apprenticeship training and supervision by the project employed Registered Psychological Counsellor. |
| **7. Where** | The face-to-face educational outreach sessions took place at the district health offices. The *in vivo* apprenticeship training took place on-site at the clinics where counsellors were based. |
| **8. When and how much** | HIV lay-counsellors received a group, structured face-to-face educational outreach over three days. This was followed by *in vivo* apprenticeship training at the clinics where the supervisor observed three sessions facilitated by the counsellors. Supervision was provided monthly and on an ad hoc basis depending on nee. |
| **9. Tailoring** | None |
| **10. Modifications** | None |
| **11. How well planned** | Facilities were mandated to select HIV counsellors who would be capacitated to provide the counselling in each facility. They were supported by the District Training Coordinator. |
| **12. How well actual** | Out of a total 19 facilities, 20 HIV counsellors were capacitated to deliver the counselling service. |
